# ClAg_14_(C≡C^t^Bu)_12_ Nanoclusters as Efficient and Selective Electrocatalysts Toward Industrially Relevant CO_2_ Conversion

**DOI:** 10.1002/advs.202306089

**Published:** 2023-12-25

**Authors:** Hoeun Seong, Kiyoung Chang, Fang Sun, Sojung Lee, Sang Myeong Han, Yujin Kim, Chang Hyuck Choi, Qing Tang, Dongil Lee

**Affiliations:** ^1^ Department of Chemistry Yonsei University Seoul 03722 Republic of Korea; ^2^ School of Chemistry and Chemical Engineering Chongqing Key Laboratory of Theoretical and Computational Chemistry Chongqing University Chongqing 401331 China; ^3^ Department of Chemistry Pohang University of Science and Technology (POSTECH) Pohang 37673 Republic of Korea; ^4^ Institute for Convergence Research and Education in Advanced Technology (I–CREATE) Yonsei University Seoul 03722 Republic of Korea

**Keywords:** alkynyl ligand, CO_2_ electroreduction, gas flow cell, silver cluster, zero‐gap electrolyzer

## Abstract

Atomically precise metal nanoclusters (NCs) have emerged as a promising frontier in the field of electrochemical CO_2_ reduction reactions (CO_2_RR) because of their distinctive catalytic properties. Although numerous metal NCs are developed for CO_2_RR, their use in practical applications has suffered from their low‐yield synthesis and insufficient catalytic activity. In this study, the large‐scale synthesis and electrocatalytic performance of ClAg_14_(C≡C^t^Bu)_12_^+^ NCs, which exhibit remarkable efficiency in catalyzing CO_2_‐to‐CO electroreduction with a CO selectivity of over 99% are reported. The underlying mechanisms behind this extraordinary CO_2_RR activity of ClAg_14_(C≡C^t^Bu)_12_^+^ NCs are investigated by a combination of electrokinetic and theoretical studies. These analyses reveal that different active sites, generated through electrochemical activation, have unique adsorption properties for the reaction intermediates, leading to enhanced CO_2_RR and suppressed hydrogen production. Furthermore, industrially relevant CO_2_‐to‐CO electroreduction using ClAg_14_(C≡C^t^Bu)_12_^+^ NCs in a zero‐gap CO_2_ electrolyzer, achieving high energy efficiency of 51% and catalyst activity of over 1400 A g^−1^ at a current density of 400 mA cm^−2^ is demonstrated.

## Graphical abstract

The large‐scale synthesis of atomically precise ClAg_14_(C≡C^t^Bu)_12_ nanoclusters is demonstrated, achieving a high yield of 90% on a scale of over 10 g. These nanoclusters exhibit extraordinary electrocatalytic activity for CO_2_ electroreduction to CO with a CO selectivity exceeding 99% and an energy efficiency of 51% at 400 mA cm^−2^, demonstrating a remarkable catalyst activity of over 1400 A g^−1^.

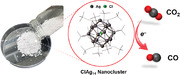

## 1 Introduction

The electrochemical CO_2_ reduction reaction (CO_2_RR) is widely recognized as a promising approach for the sustainable conversion of CO_2_ into valuable chemicals.^[^ ^1^ ^]^ Among the various CO_2_RR products, CO holds particular economic appeal due to its favorable market price^[^ ^2^ ^]^ and extensive applications in various industries, such as Fischer–Tropsch synthesis and polyketone fabrication.^[^ ^3^ ^]^ Despite significant progress in the development of efficient electrocatalysts for CO_2_ reduction,^[^ ^4^ ^]^ meeting the industrial benchmarks for CO_2_‐to‐CO electrolyzers remains challenging, including achieving current densities (*j*) exceeding 400 mA cm^−2^ with >95% Faradaic efficiency for CO production (FE_CO_) at cell potentials of <2.5 V.^[^ ^5^ ^]^ In typical H‐shaped electrolytic cells, current densities are limited to tens of milliamperes^[^ ^1^, ^6^ ^]^ due to the low solubility of CO_2_ in aqueous electrolyte solutions.^[^ ^7^ ^]^ To overcome the mass transport limitations of CO_2_, gas diffusion electrode (GDE)‐based flow electrolyzers have been developed.^[^ ^7^ ^]^ Furthermore, membrane electrode assembly (MEA)‐based zero‐gap electrolyzers have been recently introduced to minimize energy losses from the ohmic drop.^[^ ^8^ ^]^ However, commercial Ag nanoparticles (NPs) employed in the zero‐gap electrolyzers exhibit low material utilization and energy efficiency, emphasizing the need for commercially viable electrocatalysts with high mass activity and energy efficiency.

In the past decade, atomically precise metal nanoclusters (NCs) have emerged as promising catalysts for CO_2_ electroreduction due to their unique electrocatalytic activities.^[^ ^9^ ^]^ The first breakthrough came in 2012, with the utilization of Au_25_(SR)_18_ NCs, where SR represents a thiolate ligand, as an efficient electrocatalyst toward CO_2_ conversion in an H‐shaped electrolyzer.^[^ ^10^ ^]^ Since then, numerous Au‐based NCs have been developed as CO_2_RR electrocatalysts by controlling their composition^[^ ^11^ ^]^ and structure.^[^ ^12^ ^]^ Furthermore, Ag‐based NCs^[^ ^13^ ^]^ and Cu‐based NCs^[^ ^14^ ^]^ have been explored as electrocatalysts for CO_2_RR, selectively producing CO and HCOO^−^, respectively, as the primary products in H‐type electrolyzers. However, the current densities achieved for CO_2_RR in the H‐type electrolyzers have remained below 50 mA cm^−2^ due to CO_2_ mass transport limitations.

Recently, we demonstrated commercially viable CO_2_‐to‐CO electroreduction using Au_25_ NCs, achieving a CO current density (*j*_CO_) of 540 mA cm^−2^ at a modest cathode overpotential (*η*) of 0.7 V in a gas flow electrolyzer.^[^ ^15^ ^]^ Moreover, we achieved a CO_2_‐to‐CO conversion energy efficiency of 57% at 200 mA cm^−2^ by employing active site‐engineered AuAg_12_Au_12_ NCs in a zero‐gap CO_2_ electrolyzer with minimized cell resistance.^[^ ^4j^ ^]^ Although certain Au‐based NCs exhibit remarkable CO_2_RR activities, large‐scale synthesis of these NCs remains challenging. Facile and high‐yield synthesis methods are essential for the practical application of metal NCs in commercially relevant CO_2_RR processes.

Here, we report efficient and selective CO_2_‐to‐CO electroreduction catalyzed by ClAg_14_(C≡C^t^Bu)_12_^+^ NCs (referred to as ClAg_14_ NCs). We have achieved the stoichiometric synthesis of atomically precise ClAg_14_ NCs at a scale exceeding 10 g with a yield of 90%. Electrocatalytic investigation revealed that the ClAg_14_ NCs exhibited extraordinary catalytic activity and near‐unity selectivity for CO production. Density functional theory (DFT) calculations supported that the high CO selectivity of the ClAg_14_ NCs originates from their optimal binding properties, favoring CO_2_RR over hydrogen evolution reaction (HER). Finally, we demonstrated highly energy‐efficient CO_2_‐to‐CO electroreduction in a zero‐gap CO_2_ electrolyzer equipped with the ClAg_14_ NCs.

## 2 Experimental Section

### 2.1 Chemicals

Silver nitrate (AgNO_3_, >99.9%), Silver tetrafluoroborate (AgBF_4_, 99%), 3,3‐dimethyl‐1‐butyne (HC≡C^t^Bu, 98%), tetrabutylammonium chloride (Bu_4_NCl, 95%), tetrabutylammonium fluoride (Bu_4_NF, 99%), tetrabutylammonium bromide (Bu_4_NBr, >99%), potassium hydroxide (KOH, >85%), and potassium chloride (KCl, >99%) were purchased from Alfa Aesar. Triethylamine (Et_3_N, >99%), sodium borohydride (NaBH_4_, 99%), tetraphenylphosphonium bromide (PPh_4_Br, 97%), and deuterium oxide (D_2_O, 99.9 at% D) were purchased from Merck. 2,4‐dimethylbenzenethiol (HSPhMe_2_, >96%) was purchased from Tokyo Chemical Industry. Extra‐pure grade methanol, *n*‐pentane, *n*‐hexane, diethyl ether, dichloromethane (CH_2_Cl_2_), and tetrahydrofuran (THF) were used. Water was purified using a Millipore Milli‐Q system (DI water, 18.2 MΩ cm). All chemicals were used as received without further purification.

### 2.2 Synthesis of Silver NCs

ClAg_14_(C≡C^t^Bu)_12_^+^ (denoted as ClAg_14_) NCs, where C≡C^t^Bu is 3,3‐dimethyl‐1‐butynyl, were synthesized following a previously reported procedure.^[^ ^16^ ^]^ Briefly, AgBF_4_ (100 mg, 0.51 mmol) was dissolved in THF (2 mL) containing HC≡C^t^Bu (0.054 mL, 0.44 mmol), Bu_4_NCl (10 mg, 0.036 mmol), and Et_3_N (0.08 mL, 0.52 mmol). Subsequently, the solution was vigorously stirred for 4 h. Following the reaction, the product was washed with copious amounts of DI water and diethyl ether. Finally, the product was purified via crystallization by layering diethyl ether on the NC solution in CH_2_Cl_2_. This synthesis was scaled up by using 15 g of AgBF_4_ salt and other reactants in the same proportion. In this large‐scale synthesis, the product was also purified via crystallization, which ensured that the product was the highly pure single crystals of ClAg_14_(C≡C^t^Bu)_12_^+^ NCs and contained no other sized NCs. The large‐scale synthesis of ClAg_14_(C≡C^t^Bu)_12_^+^ NCs was very reproducible with 90 ± 2% yield based on the Ag atom (averaged out of three independent syntheses). FAg_14_(C≡C^t^Bu)_12_^+^ and BrAg_14_(C≡C^t^Bu)_12_^+^ NCs were similarly synthesized using equivalent moles of Bu_4_NF and Bu_4_NBr, respectively, instead of Bu_4_NCl in the synthetic method aforementioned. The typical yields of these NCs were 85–90%.

Hollow Ag_14_(C≡C^t^Bu)_12_^2+^ NCs were prepared according to the synthetic method reported elsewhere.^[^ ^17^ ^]^ AgBF_4_ (100 mg, 0.51 mmol) was dissolved in DI water (5 mL), and HC≡C^t^Bu (0.054 mL, 0.44 mmol) was added to the solution. After vigorously stirring for 12 h, the NC product was precipitated out of the solution. The product was thoroughly washed with DI water, diethyl ether, and *n*‐pentane, and finally purified by crystallization. The typical yield was ≈60%.

Ag_25_(SPhMe_2_)_18_ (denoted as Ag_25_), where SPhMe_2_ is 2,4‐dimethylbenzenthiolate, was prepared following a previously reported procedure.^[^ ^18^ ^]^

### 2.3 Characterization of NCs

Ultraviolet–visible (UV–vis) absorption spectra of the NCs in CH_2_Cl_2_ were obtained using a Shimadzu UV–Vis–NIR spectrophotometer (UV‐3600). ESI mass spectra were acquired using the positive ion mode of an electrospray ionization (ESI) mass spectrometer (Agilent 6230 TOF LC/MS). Thermogravimetic analysis (TGA) was performed on a Q50 thermal analyzer (TA Instruments) from 70 to 1000 °C at a heating rate of 10 °C min^−1^ under air atmosphere. The powder X‐ray diffraction (XRD) pattern was obtained using a Rigaku Ultima IV setup equipped with a graphite‐monochromated Cu Kα radiation source (40 kV, 40 mA). The simulated XRD pattern was extracted through Mercury (Ver. 2020.1) software based on the single crystal structure. Transmission electron microscopy (TEM) images were obtained using an aberration‐corrected transmission electron microscope (JEM‐ARM200F NEOARM, JEOL). ClAg_14_‐containing GDEs (ClAg_14_/GDEs) before and after the activation process were crushed and dispersed in CH_2_Cl_2_. The suspension was drop cast onto a 400 mesh Formvar/carbon‐coated copper grid (01814‐F, Ted Pella) and dried for 1 h at room temperature before imaging. Square wave voltammogram (SWV) was obtained using an electrochemical workstation (660D, CH Instruments). A CH_2_Cl_2_ solution containing NCs (1.0 mm) and a supporting electrolyte of 0.1 m tetrabutylammonium hexafluorophosphate (Bu_4_NPF_6_) was purged with high‐purity Ar gas to completely remove O_2_ from the solution. Pt disk electrodes (0.40 mm diameter) were used as the working and counter electrodes, and Ag wire was used as the quasi‐reference electrode. After obtaining SWVs, the potentials were calibrated using ferrocene (Fc^+/0^) as an internal reference. The SWV scan rate was 100 mV s^−1^ with a pulse height and width of 20 mV and 20 ms, respectively.

Single crystals of ClAg_14_(C≡C^t^Bu)_12_^+^ NCs were grown for 3 d at 25 °C by layering diethyl ether over the CH_2_Cl_2_ solution containing ClAg_14_(C≡C^t^Bu)_12_^+^ NCs. The data collection proceeded using the PAL BL2DSMDC program.^[^ ^19^ ^]^ Cell refinement, reduction, and absorption correction were conducted using HKL3000sm (Version 716.7).^[^ ^20^ ^]^ The crystal structure of ClAg_14_(C≡C^t^Bu)_12_^+^ NCs was solved using the direct method with SHELX‐XT (Ver. 2014/5)^[^ ^21^ ^]^ and refined by full‐matrix least‐squares calculations with the SHELX‐XL (Ver. 2016/4)^[^ ^22^ ^]^ in the Olex2^[^ ^23^ ^]^ program package.

### 2.4 Characterization of Activated NCs

Electrochemically activated ClAg_14_ NCs were prepared according to the previously reported procedure.^[^ ^12b^ ^]^ Briefly, the activated ClAg_14_ NCs were obtained after the electrochemical activation process. Due to the strong hydrophobic interaction between alkynyl ligands of the ClAg_14_ NC and GDE (W1S1011, Ce‐Tech), the recovery of the electrochemically activated ClAg_14_ NCs from the GDE was very low. Therefore, a nickel foam (NF, 29‐04275‐01, Invisible Inc.) substrate was used instead. To ensure the uniform activation of the ClAg_14_ NCs, a very thin layer of ClAg_14_ NCs (2 nmol cm^−2^) was uniformly drop cast onto a large‐area NF (100 cm^2^) and subsequently activated using CPE at −0.96 V versus reversible hydrogen electrode (RHE) for 1 h. After the activation process, the electrode was thoroughly washed with DI water and diethyl ether to remove the remaining electrolyte salts and alkynyl ligands, respectively, and then the activated ClAg_14_ NCs were repeatedly retrieved using CH_2_Cl_2_ (10 mL x 3). The recovery yield of the activated ClAg_14_ NCs was over 90%. The electrochemically activated ClAg_14_ NCs exhibited nearly identical CO_2_RR activity to that of the activated ClAg_14_/GDE (Figure S7, Supporting Information), validating the activation process on the NF.

Extended X‐ray absorption fine structure (EXAFS) analysis was conducted for the characterization of the pristine and activated ClAg_14_ NCs. EXAFS data for the ClAg_14_ NCs over the Ag K‐edge were collected in transmission mode using ionization detectors (Oxford) at PAL (7D‐XAFS beamline). The beam energy, ring current, step, and duration time were 2.5 GeV, 200 mA, 0.03 Å^−1^, and 3 s, respectively. ClAg_14_ NCs (≈10 mg) were mounted in a sample holder (3 × 8 × 2 mm). The EXAFS data were calibrated using metallic Ag foils before and after measurement, and the many‐body attenuation factors (*S*_0_^2^) were determined as 0.8. Artemis implemented in the Demeter program package (0.9.26) was exploited after the data processing in Athena.^[^ ^24^ ^]^ Amplitudes and phase shifts were calculated using FEFF7.

### 2.5 CO_2_ Electroreduction Experiments

The CO_2_RR activity of ClAg_14_ NCs was evaluated in a gas flow cell. GDEs consisting of a gas diffusion layer (GDL, 360 µm‐thick) and a microporous layer (MPL, 50 µm‐thick) were used as a support of the NCs. The CO_2_RR activity of ClAg_14_ NCs was compared with those of Ag_25_ NCs and commercial Ag NPs(Dioxide Materials), which had been evaluated before in a similar electrolyzer system.^[^ ^4j^ ^]^ The ligand‐protected NCs can be directly immobilized in the MPL of GDE.^[^ ^12b^ ^]^ More specifically, the catalyst solution was prepared by dissolving a predetermined amount of NCs in 1:1 (v/v) mixture of CH_2_Cl_2_‐acetone (0.32 mL). The catalyst solution was then drop cast onto the MPL side of GDE (2.5 × 2.5 cm^2^) and dried under room condition over 1 h. The atom‐precise Ag NCs enabled precise control of their loading at the molecular level. In a previous study,^[^ ^4j^ ^]^ the optimal loading of Ag_25_ NCs was determined to be 10.6 nmol cm^−2^. Therefore, the CO_2_RR activities of Ag_25_ and ClAg_14_ NCs were compared at the same loading (10.6 nmol cm^−2^). The loading of the commercial Ag NPs on GDE was 4.8 mg cm^−2^ and used as received. In this experiment, it was found that the CPE condition (−0.96 V for 1 h) was the optimal activation condition for both Ag_25_ and ClAg_14_ NCs. Both NCs were electrochemically activated prior to electrocatalysis experiments. The AgNP/GDE did not show any change after CPE (at −0.96 V for 1 h) and thus was used without electrochemical treatment. The CO_2_RR performance of Ag catalysts was evaluated in a laboratory‐made gas flow cell^[^ ^4j^ ^]^ comprised of an NC/GDE cathode (2 cm^2^), an NF anode (2 cm^2^), and an anion exchange membrane (AEM, Fumasep, FAAM‐40, FuMA‐Tech) placed between the two compartments. A Ag/AgCl (1.0 m KCl) reference electrode was positioned in the cathode compartment. CO_2_ gas (20 mL min^−1^) was supplied to the GDL side of the cathode, and the fresh electrolyte (1 mL min^−1^) was circulated on the MPL side of the cathode and frontside of the anode. For HER experiments, Ar gas (20 mL min^−1^) was supplied to the GDL side instead of CO_2_ gas.

The full‐cell measurements for the CO_2_‐H_2_O co‐electrolysis were carried out in a zero‐gap CO_2_ electrolyzer (Figure 5a), comprised of an NC/GDE cathode (1 cm^2^), an NF anode (1 cm^2^), and an AEM (Sustainion X37‐50, RT grade, Dioxide Materials). 5 cm^2^ electrodes were used for the single‐pass conversion efficiency (SPCE) measurements. CO_2_ gas (200 mL min^−1^, humidified at 25 °C) was supplied to the cathode via serpentine flow field, and the fresh 1.0 m KOH electrolyte solution (3 mL min^−1^) was supplied to the anode chamber.

The electrochemical experiments, including constant potential electrolysis (CPE), linear sweep voltammetry, electrochemical impedance spectroscopy (EIS), and galvanostatic electrolysis were conducted using a potentiostat (ZIVE BP2, WonATech). The electrochemical experiments were conducted and averaged out of 2,3 independent experiments. The electrochemical experiments were very reproducible with the precise loading control of NCs at the molecular level and the error bars were actually smaller than the symbol size in the graphs. For the comparison of catalytic activity, the cathodic potentials were iR‐corrected in some figures, which was pointed out in the figure captions (unless otherwise specified, the potentials are the applied potentials without iR correction). The produced gas products were quantified using in‐line gas chromatography (Agilent, GC 7890B) equipped with a thermal conductivity detector (TCD) and a flame ionization detector (FID). Liquid products were not observed in any CO_2_RR experiments on NC/GDEs or AgNP/GDE.

To compare the CO_2_RR activity of ClAg_14_ NCs with those of other efficient metal NC catalysts, the CO_2_RR activity of ClAg_14_ NCs was also evaluated in a laboratory‐designed H‐cell constructed by assembling two glass compartments separated by a Nafion 117 membrane. The ClAg_14_/GDE (1 cm^2^) and Pt foil (1 cm^2^) were used as the cathode and anode, respectively. The Ag/AgCl reference electrode (1.0 m KCl) was equipped in the cathode compartment. A mixed aqueous solution of 0.05 m KHCO_3_ and 1.0 m KCl was used as an electrolyte solution. Before measuring the electrocatalytic activity of ClAg_14_/GDE for CO_2_RR, the catholyte solution was purged with CO_2_ gas for 1 h to make a CO_2_‐saturated solution, and then tightly sealed to prevent the leakage of gaseous molecules. The CPE experiments were performed on a potentiostat (ZIVE BP2, WonATech) for 1 h at each potential with vigorous stirring of the catholyte solution to continuously supply CO_2_ to the cathode. After the CPE experiments, the headspace of the cathode compartment was analyzed using gas chromatography (Agilent, GC 7890B) equipped with a TCD and a FID.

### 2.6 Operando Infrared (IR) Measurements

Operando attenuated total reflection Fourier transform infrared (ATR‐FTIR) experiments were performed in a laboratory‐made H‐type cell equipped with a Ge horizontal ATR crystal (Pike Technologies) assembled on an FTIR spectrophotometer (Thermo Fisher Scientific, Nicolet iS50) equipped with a mercury cadmium telluride detector. The electrochemical cell was filled with 1.0 m NaClO_4_ electrolyte solution (30 mL) and purged with CO_2_ for 30 min before conducting operando experiments during which CO_2_ gas was continuously supplied to the solution. NaClO_4_ electrolyte was used instead of bicarbonate‐based electrolyte to minimize interference on the (bi)carbonate‐related peaks. The ClAg_14_/GDE (7 × 0.5 cm^2^) activated by CPE (at −0.96 V for 1 h) in 1.0 m KOH prior to the operando FTIR experiments was used as the working electrode. The GDE was mounted on the ATR crystal with the MPL side facing down the crystal. A Pt foil (2 cm^2^) was used as the counter electrode and a Ag/AgCl (1.0 m KCl) electrode was used as the reference electrode. CPE experiments were conducted at −1.8 V versus standard hydrogen electrode (SHE) in CO_2_‐saturated 1.0 m NaClO_4_ solution (pH 4.5), with the vibrational modes of reaction intermediates on the ClAg_14_/GDE simultaneously monitored on the second timescale. All the spectra were corrected by subtracting the background spectrum measured before applying the CPE potential.

### 2.7 Computational Methods

The spin‐polarized DFT calculations on the electrochemical CO_2_RR and HER catalyzed by alkynyl‐protected ClAg_14_ NCs were performed using the Vienna ab initio simulation package (VASP5.4.4)^[^ ^25^ ^]^ To save computational time, the ─C≡CR group with the ─C≡C─CH_3_ ligand to mimic the ligand fragment was replaced. The ClAg_14_ NCs were put in a cubic box (18 Å × 18 Å × 18 Å), and their structures were optimized by the Perdew–Burke–Ernzerhof (PBE) function form of the generalized gradient approximation (GGA) to represent the interactions of electron exchange‐correlation.^[^ ^26^ ^]^ The projector‐augmented‐wave (PAW) method was carried out to describe the ion‐electron interactions^[^ ^27^ ^]^ and the wave functions of all the computations were expanded via a plane‐wave cutoff energy of 400 eV. The convergence criteria for energy and force were set as 10^−5^ eV and 0.02 eV A^−1^ for maximal displacement, respectively. the Brillouin zone using only Γ point and accounted for van der Waals interactions using the empirical density functional dispersion (DFT‐D3) method was sampled.^[^ ^28^ ^]^ All atoms were unconstraint and fully relaxed during the simulation.

The free energy diagrams of the CO_2_RR can be calculated according to the computational hydrogen electrode (CHE) model^[^ ^29^ ^]^ developed by Norskov et al. as follows:

(1) $$\Delta G=\Delta E+\Delta E_{\mathrm{sol}}+\Delta ZPE-T\Delta S$$

where Δ*E*, Δ*ZPE*, and Δ*S* are the changes in the total energy that can be directly obtained from DFT calculations, the difference of zero‐point energy, and the change of entropy at 298.15 K, respectively. The Δ*E*_sol_ represents a solvation correction. For *COOH and *CO, the solvation correction is −0.25 eV and −0.1 eV, respectively.^[^ ^30^ ^]^ For the gas molecules, the entropy is taken from the NIST database. **Table** **1** below provides the zero‐point energy and entropy correction at 298.15 K for various gas‐phase species.

**Table 1:** Zero‐point energy (ZPE) and entropy correction (‐T*S) for various gas‐phase species at *T* = 298.15 K.

Species	*ZPE* [eV]	−T*S [eV]
**CO_2_**	0.31	−0.66
**CO**	0.13	−0.61
**H_2_**	0.27	−0.40
**H_2_O**	0.59	−0.58

For the adsorbed intermediates, only vibrational entropy is considered, which is calculated from the DFT‐calculated vibrational frequencies. In addition, due to the inaccurate description of CO_2_ and CO molecules by PBE functional,^[^ ^26^, ^31^ ^]^ a correction of −0.34 eV for CO and +0.10 eV for CO_2_ was added.^[^ ^14a^ ^]^ Of note, it is often difficult to calculate the free energy of the liquid phase using the standard DFT method. Therefore, the DFT‐calculated free energy of the gas‐phase H_2_O, which is based on the free energy difference between the liquid and gas‐phase formations obtained in the NIST database was corrected:

(2) $$G\left(H_{2}O,\;l\right)-G\left(H_{2}O,g\right)=-0.09\;\mathrm{eV}$$

Therefore, a correction of −0.09 eV for H_2_O was used.

## 3 Results and Discussion

### 3.1 Large‐Scale Synthesis of ClAg_14_ NCs

**Figure 1:**
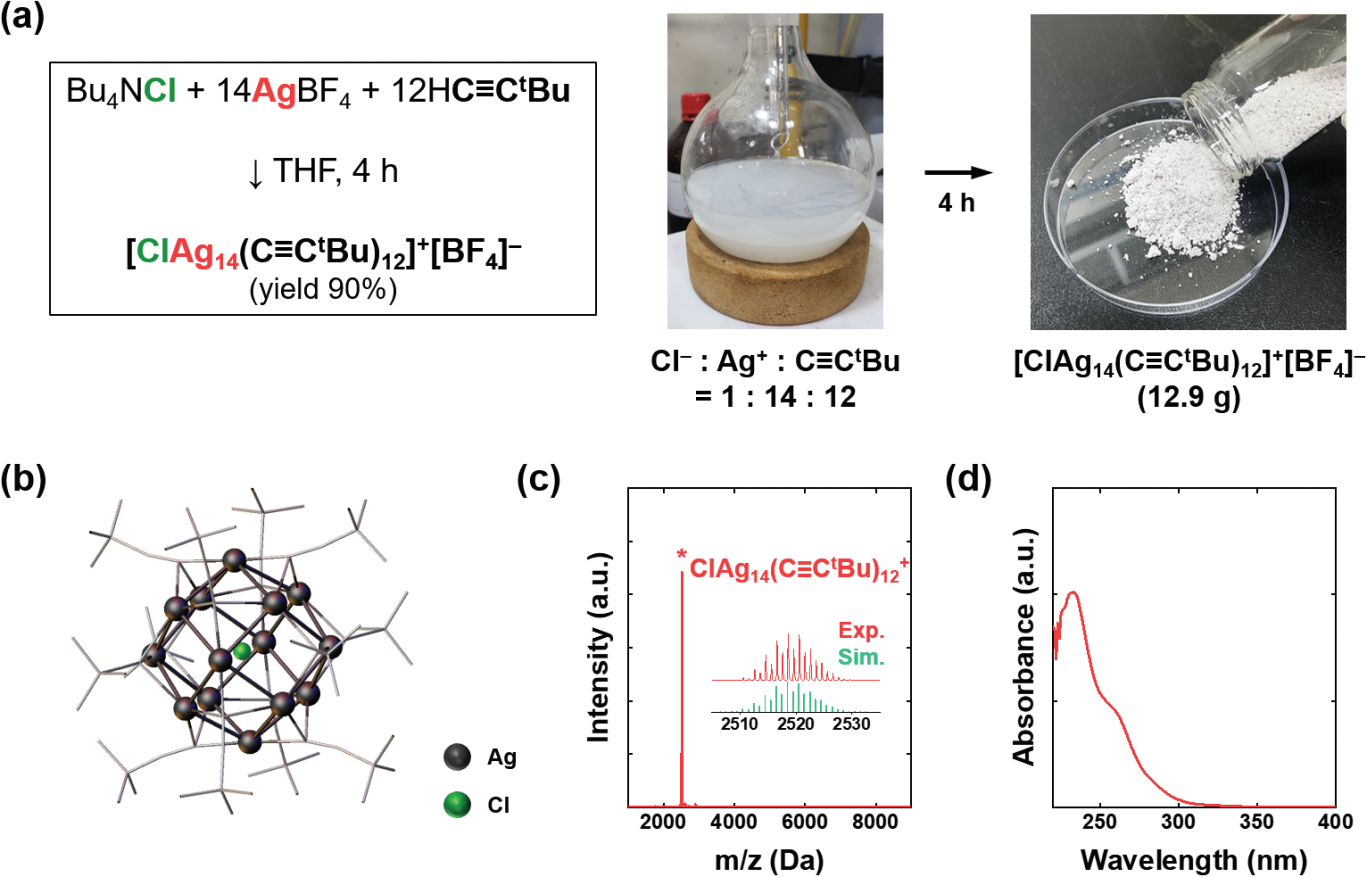
a) Chemical equations (left) and digital photographs (right) of the ClAg_14_(C≡C^t^Bu)_12_^+^ synthesis. b) Crystal structure of the ClAg_14_(C≡C^t^Bu)_12_^+^ NC (redrawn from^[^ ^16^ ^]^). All C atoms are displayed in the wireframe (H atoms are omitted for clarity). c) Positive‐mode ESI mass spectrum and d) UV–vis absorption spectrum of a CH_2_Cl_2_ solution of the ClAg_14_(C≡C^t^Bu)_12_^+^ NCs. The inset in (c) compares the experimentally obtained (red line) with the simulated isotope patterns (green bars).

**Figure** **1**a illustrates the synthetic procedure of the ClAg_14_(C≡C^t^Bu)_12_^+^ NCs (details provided in the Experimental Section). The ClAg_14_ NC catalyst was synthesized using a previously reported one‐pot procedure.^[^ ^16^ ^]^ At the early stage of the synthesis, a series of silver–alkynyl oligomers formed, which subsequently transformed into a monodisperse Ag_14_(C≡C^t^Bu)_12_ cage compound templated by Cl^−^ anion through the argentophilic interaction.^[^ ^16^, ^32^ ^]^ The ClAg_14_ NCs could be synthesized stoichiometrically (i.e., Cl^−^: Ag^+^: C≡C^t^Bu = 1: 14: 12), resulting in a high synthetic yield. For example, 12.9 g (4.96 mmol) of ClAg_14_ NCs was obtained from 15.0 g (77.1 mmol) AgBF_4_ precursor, yielding 90 ± 2% based on the Ag atom. Notably, the one‐pot synthesis allowed for producing over 10 g of ClAg_14_ NCs within 4 h, facilitating the scale‐up of the synthetic process. The templating of the Ag_14_(C≡C^t^Bu)_12_ cage could also be achieved with other halogen anions such as F^−^ and Br^−^ (Figure S1, Supporting Information). Moreover, the hollow Ag_14_(C≡C^t^Bu)_12_ cage could be synthesized without the templating anion, whereas the synthetic yield was significantly lower (60%). In the rest of this paper, we will focus on the ClAg_14_(C≡C^t^Bu)_12_ NCs that can be prepared with the highest synthetic yield among the NCs.

The single crystal XRD analyses revealed that the synthesized ClAg_14_ NCs had an identical structure to the previously reported^[^ ^16^ ^]^ ClAg_14_(C≡C^t^Bu)_12_^+^ composed of a Cl center surrounded by an Ag_8_ cube and an Ag_6_ octahedral cage (Figure 1b; Figure S2, Supporting Information). The Cl@Ag_14_ framework is further protected with 12 alkynyl ligands. Each of the Ag atoms in the Ag_8_ framework is coordinated with three ligands, while the Ag atoms in the Ag_6_ cage are linearly bonded with two ligands. The synthesized ClAg_14_ NCs were further characterized by ESI mass spectrometry (ESI‐MS). The ESI‐MS displayed a highly intense single peak at m/z = 2518 Da, corresponding to the ClAg_14_(C≡C^t^Bu)_12_^+^ cation (Figure 1c). The isotope pattern observed in the spectrum matched the simulated isotope pattern of ClAg_14_(C≡C^t^Bu)_12_^+^ (Figure 1c, inset), confirming the accurate composition of the synthesized product. Furthermore, TGA confirmed the composition of the synthesized cluster (Figure S3, Supporting Information). Notably, the molecularly pure ClAg_14_ NCs with a uniform composition and structure were synthesized over a 10 g scale. The UV–vis absorption spectrum (Figure 1d) displayed a characteristic absorption peak at 233 nm, accompanied by a shoulder peak at 269 nm. In addition, the powder XRD pattern of the cluster product synthesized over a 10 g scale closely matched with the simulated diffractogram based on the crystal structure (Figure S4, Supporting Information), confirming the phase purity of the bulk of the cluster sample.

### 3.2 Catalyst Activation

**Figure 2:**
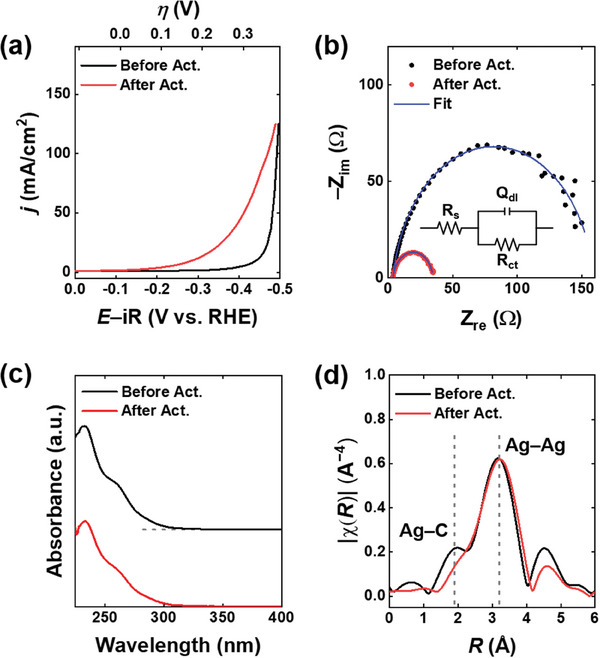
a) LSVs recorded at 20 mV s^−1^and b) Nyquist plots measured at −0.16 V on ClAg_14_ NC/GDE before and after activation. c) UV−vis absorption spectra and d) Ag K‐edge *R* space EXAFS spectra of the ClAg_14_ NCs before and after activation. The equivalent circuit^[^ ^33^ ^]^ used to fit the EIS spectra is illustrated in the inset of b). *R*_s_, solution resistance; *R*_ct_, charge‐transfer resistance; *Q*_dl_, constant phase element for double layer. All the electrochemical experiments in this figure were conducted in a CO_2_‐fed flow electrolyzer with a flowing 1.0 m KOH electrolyte solution. The potentials in panel (a) were iR‐corrected.

The CO_2_RR activity of the ClAg_14_ NCs was evaluated using a GDE‐based CO_2_‐fed flow electrolyzer, employing a circulating 1.0 m KOH electrolyte solution (details provided in the Experimental Section). In a recent study, we have demonstrated that the thiolate‐protected metal NCs can undergo electrochemical activation by partially losing their ligands.^[^ ^11^, ^12^ ^]^ Those results raised the question of whether the alkynyl‐protected ClAg_14_ NCs can undergo similar electrochemical activation processes. To investigate this theory, we compared several electrocatalytic properties of the ClAg_14_ NCs before and after electrochemical treatment. The ClAg_14_/GDEwas subjected to CPE at −0.96 V versus RHE (hereafter, all the potentials are against RHE unless stated otherwise) for 1 h. The resulting linear sweep voltammograms (LSVs) (**Figure** **2**a) clearly indicated a significant increase in the current response of the ClAg_14_ NCs after experiencing reductive potentials, suggesting successful electrochemical activation of the NCs. The *j*_CO_ values, determined through CPE experiments, also exhibited an increase for the activated NCs (Figure S5, Supporting Information).

The electrochemical activation of the ClAg_14_ NCs was further confirmed by EIS. Figure 2b illustrates the Nyquist plots, which exhibited distinct differences before and after activation. Fitting of the plots allowed the determination of the charge‐transfer resistance (*R*_ct_) value, which significantly decreased from 155 to 31 Ω upon activation. In contrast, the solution resistance (*R*_s_) value remained unchanged (Figure S6, Supporting Information). These results provided additional evidence that the electrochemical treatment successfully activated the ClAg_14_ NCs, resulting in a significant reduction in the *R*_ct_.

To investigate the structural changes of the activated ClAg_14_ NCs, we retrieved the NCs from the electrode following the CPE experiments. To increase the recovery of the activated NCs, we used a NF substrate instead of the GDE (details provided in the Experimental Section). The CO_2_RR activity of the ClAg_14_ NCs activated on the NF was nearly identical to that of ClAg_14_/GDE, validating this activation procedure (Figure S7, Supporting Information). The UV–vis absorption spectrum of the activated ClAg_14_ NCs displayed no significant differences compared to the pristine ClAg_14_ NCs (Figure 2c), indicating that the overall structure of the ClAg_14_ NCs was retained after electrochemical activation. TEM images of the ClAg_14_/GDE showed similar size distribution before and after electrochemical activation (Figure S8, Supporting Information), indicating that the NCs were stable during the activation process.

EXAFS spectroscopy analysis was performed to determine the structure of the activated ClAg_14_ NCs. The Ag *K*‐edge EXAFS spectrum of the pristine ClAg_14_ NCs displayed a prominent peak at 3.2 Å, attributed to the Ag─Ag bond, along with additional Ag─C scattering at 1.9 Å (Figure 2d). Following activation, the Ag─Ag bond remained unchanged, while the Ag─C peak significantly decreased, as shown in Figure 2d, suggesting the removal of alkynyl ligands from the ClAg_14_ NCs. The atomic structure of the activated ClAg_14_ NCs was determined by fitting the Ag K‐edge EXAFS spectrum of electrochemically treated NCs (Figure S9, Supporting Information). The coordination number (CN) of the Ag─C bond decreased from 12/14 to 8/14 after activation (Table S1, Supporting Information), indicating the removal of approximately four ligands per NC [i.e., ClAg_14_(C≡C^t^Bu)_8_]. Additionally, the Ag─Ag bond length was slightly shortened after de‐ligation (Table S1, Supporting Information). The EXAFS analysis confirmed that the alkynyl‐protected NCs can be electrochemically activated by removing several of their alkynyl ligands. The ClAg_14_ NCs were electrochemically activated on GDE prior to all electrocatalysis experiments. The Ag sites exposed by ligand removal in the activated ClAg_14_ NCs serve as the active sites for CO_2_RR (vide infra).

### 3.3 CO_2_RR Performance in a Gas Flow Cell

**Figure 3:**
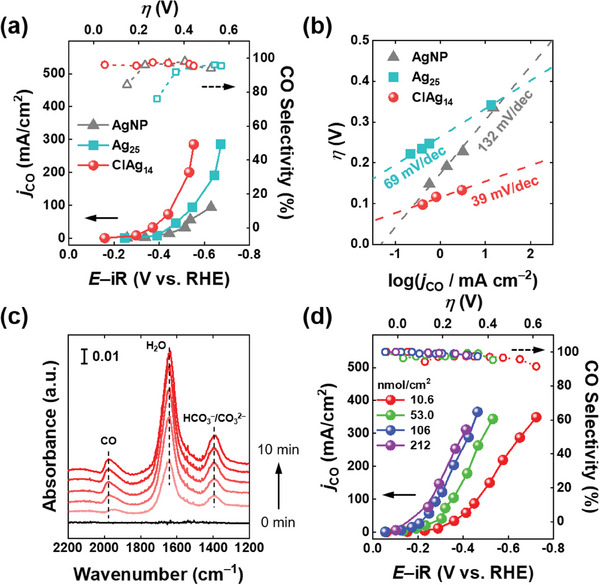
a) *j*_CO_ and the CO selectivity of various Ag catalysts. Note that the loadings of the Ag NCs on GDE were equal to 10.6 nmol cm^−2^, while the Ag NPs loading on GDE was 4.8 mg cm^−2^. b) Tafel plots constructed for CO_2_‐to‐CO electroreduction of various Ag catalysts. c) Temporal evolution of IR spectra obtained on ClAg_14_/GDE during CPE experiment at −1.8 V versus SHE for 10 min in CO_2_‐saturated 1.0 m NaClO_4_ solution. d) ClAg_14_ NC loading effect on the CO_2_RR performance. All the experiments in this figure were conducted in a CO_2_‐fed flow electrolyzer with a flowing 1.0 m KOH electrolyte solution. In panels (a), (b), and (d), the potentials were iR‐corrected and the errors lie within the symbols in the graphs.

Next, we evaluated the CO_2_RR activity of the activated ClAg_14_ NCs in a gas flow cell. For comparison, we compared the CO_2_RR activity with those of previously reported^[^ ^11^, ^12^ ^]^ Ag_25_(SR)_18_, where SR = thiolate, NCs (Ag_25_ for short) and commercial Ag nanoparticles (AgNPs, Dioxide Materials). Note that Ag_25_ NCs were also electrochemically activated at −0.96 V for 1 h prior to electrocatalysis experiments. As illustrated in **Figure** **3**a, both ClAg_14_ and Ag_25_ NCs exhibited higher *j*_CO_ values compared to the AgNPs. Additionally, among the two NCs, the ClAg_14_ NCs displayed superior CO_2_RR activity when compared to the Ag_25_ NCs. The *j*_CO_ obtained with the ClAg_14_ NCs reached 285 mA cm^−2^ at *η* = 0.44 V, which was significantly higher than the *j*_CO_ of the Ag_25_ NCs (94 mA cm^−2^). While the AgNPs and Ag_25_ NCs exhibited relatively low CO selectivity (≈80%) near their respective onset potentials, the ClAg_14_ NCs demonstrated remarkable CO selectivity exceeding 95% at the entire studied potential region. Moreover, the other halogen‐centered XAg_14_ NCs (X = F, Br) and the hollow Ag_14_(C≡C^t^Bu)_12_ cage showed nearly identical *j*_CO_ values and CO selectivities (Figure S1b, Supporting Information), indicating that the influence of the center atom on the CO_2_RR activity is insignificant.

As the ClAg_14_ NCs exhibited high CO_2_RR activity, it would be instructive to compare their activity with those of other efficient metal NC catalysts. However, most of the previous studies were conducted in H‐cells and thus the CO_2_RR activities in H‐cells were compared by plotting their turnover frequencies (TOFs) as a function of the applied potential. As shown in Figure S10a and Table S2 (Supporting Information), the TOFs of the ClAg_14_ NCs were higher than those reported for the most efficient Ag NC catalysts and comparable to those obtained from Au NC catalysts in similar H‐cells. It is noteworthy that the *j*_CO_ values and CO selectivities of the ClAg_14_ NCs were particularly high in a gas flow cell (Figure S10b, Supporting Information), and thus the CO_2_RR activities of the ClAg_14_ NCs were evaluated in a gas flow cell in the rest of this paper.

Electrokinetic studies were conducted to elucidate the origin of the extraordinary CO_2_RR activity of the ClAg_14_ NCs. Tafel analyses, when conducted in the kinetically controlled region, provide insights into the reaction pathways of different catalysts. The AgNPs exhibited a Tafel slope of 132 mV dec^−1^, whereas both Ag_25_ and ClAg_14_ NCs demonstrated significantly lower values of 69 and 39 mV dec^−1^, respectively (Figure 3b). These results suggested that the CO_2_RR proceeds through different pathways on these NCs.

The electrochemical CO_2_‐to‐CO reduction in neutral–alkaline solutions can be described by the following elementary steps (where * denotes an active site) (Equations (3), (4), (5), (6), (7)):^[^ ^34^ ^]^

(3) $$*+{\mathrm{CO}}_{2}+e^{-}\to *{\mathrm{CO}}_{2}^{-}$$

(4) $$*{\mathrm{CO}}_{2}^{-}+H_{2}O\to *\mathrm{COOH}+{\mathrm{OH}}^{-}$$

(5) $$*\mathrm{COOH}+e^{-}\to *{\mathrm{COOH}}^{-}$$

(6) $$*{\mathrm{COOH}}^{-}\to *\mathrm{CO}+{\mathrm{OH}}^{-}$$

(7) ∗CO→∗+CO

Equations (3) and (5) represent the first and second electron‐transfer steps, respectively. The Tafel slopes associated with these steps are 120 and 40 mV dec^−1^, respectively. The high Tafel slope observed for the Ag NPs indicates that the first electron‐transfer step is the potential‐determining step (PDS) for the CO_2_RR on AgNPs. In contrast, the significantly lower Tafel slopes observed for the Ag_25_ and ClAg_14_ NCs suggest that the first electron‐transfer step is highly facilitated, indicating that the PDS for CO_2_RR on these NCs is the second electron‐transfer step. The relatively higher Tafel slope observed for the Ag_25_ NCs implies that the electron transfer (from the active site to the CO_2_ molecule) on the Ag_25_ NCs is not as rapid as that on the ClAg_14_ NCs. In fact, the electron‐transfer rate constants (*k*_ET_) determined from EIS experiments^[^ ^35^ ^]^ at −0.56 V followed the order AgNPs < Ag_25_ NCs < ClAg_14_ NCs, which aligns well with the trend observed in the Tafel slope analyses (Figure S11, Supporting Information). This analysis highlighted the fast electron‐transfer property of the ClAg_14_ NCs as the origin of its high CO_2_RR activity.

Water dissociation is considered another key step in CO_2_RR as water serves as the primary source of proton in neutral–alkaline media.^[^ ^36^ ^]^ To investigate whether the protonation step (Equation 4) is involved in the PDS of CO_2_RR on the ClAg_14_ NCs, we studied the kinetic isotope effect (KIE) of H/D^[^ ^34^ ^]^ on the CO_2_RR. Figure S12a (Supporting Information) demonstrates that the ClAg_14_ NCs exhibited nearly identical *j*_CO_ values in H_2_O‐ and D_2_O‐based electrolyte solutions, indicating the absence of a H/D KIE for CO_2_RR. This suggests that the CO_2_RR intermediates generated on the ClAg_14_ NCs undergo rapid protonation, and therefore the proton‐transfer step is not involved in the PDS. Interestingly, the CO selectivity was higher in a 1.0 m KOH/D_2_O solution compared to a 1.0 m KOH/H_2_O solution (Figure S12a, Supporting Information). This result indicates that the HER was suppressed in the D_2_O‐based solution (Figure S12b, Supporting Information), indicating the presence of a H/D KIE for HER, while it is absent for CO_2_RR. Collectively, these findings indicate that both the first electron‐transfer and proton‐transfer steps are highly facilitated on the ClAg_14_ NCs, resulting in CO_2_RR that is primarily gated by the second electron‐transfer step (Equation 3).

We further explored the origin of the high CO selectivity of the ClAg_14_ NCs by comparing the unperturbed HER activities of the Ag catalysts in an Ar‐fed flow electrolyzer with a supplied 1.0 m KOH electrolyte solution. As demonstrated in Figure S13 (Supporting Information), among the three Ag catalysts, the ClAg_14_ NCs required the highest overpotential to achieve the same HER current density, which explains the suppressed H_2_ production during CO_2_RR.

In order to confirm the CO_2_RR active sites of the ClAg_14_ NCs, operando ATR‐FTIR study was conducted during CO_2_RR.^[^ ^37^ ^]^ CPE was conducted at −1.8 V versus SHEin CO_2_‐saturated 1.0 m NaClO_4_ solution (pH 4.5), with the vibrational modes of reaction intermediates on the ClAg_14_/GDE simultaneously monitored on the second timescale (see the Experimental Section and Figure S14 (Supporting Information) for details). As can be seen in Figure 3c, three dominant peaks centered at 1396, 1642, and 1974 cm^−1^ grow with increasing CPE time. While the absorption bands at 1396 and 1642 cm^−1^ were assignable to (bi)carbonate ions and interfacial water, respectively,^[^ ^38^ ^]^ the band at 1974 cm^−1^ could be assigned to the *CO intermediate linearly bound to Ag surface which is typically observed between 1900 and 2000 cm^−1^.^[^ ^39^ ^]^ Operando ATR‐FTIR clearly shows that the Ag sites exposed by ligand removal serve as the active sites for the electroreduction of CO_2_ to CO.

Metal NCs composed of a few tens of metal atoms exhibit high atom efficiency for catalytic reactions.^[^ ^11c^ ^]^ ClAg_14_ NCs indeed displayed outstanding mass activities, as shown in Figure S15a (Supporting Information). The mass activities of ClAg_14_ NCs surpassed those of AgNPs and even Ag_25_ NCs. Notably, the mass activity of the ClAg_14_ NCs was remarkably high at low catalyst loadings where CO_2_RR is not limited by the CO_2_ mass transfer. At a low catalyst loading of 0.55 µg cm^−2^, the ClAg_14_ NCs displayed a remarkable mass activity of 58473 A g^−1^, surpassing AgNPs (288 A g^−1^) and Ag_25_ NCs (21154 A g^−1^). Surprisingly, the ClAg_14_ NCs maintained a CO selectivity of over 90% even at this extremely low loading, while the other Ag catalysts showed sharp reductions of CO selectivity at low catalyst loadings (Figure S15b, Supporting Information).

The ligand‐protected ClAg_14_ NCs can be directly immobilized in the MPL of the GDE. The CPE results at various ClAg_14_ NC loadings with an increasing applied potential are shown in Figure 3d. The *j*_CO_ of ClAg_14_/GDE linearly increased with increasing the NC loading and leveled off at the loading of over 106 nmol cm^−2^ (Figure S16, Supporting Information). The linear increase in the *j*_CO_ values implies that most of the deposited NCs participate in the CO_2_RR, resulting in a high mass activity for CO production. Moreover, the CO selectivity was also increased to >99% for higher ClAg_14_ NC loadings in the overpotential range of 0.1–0.4 V, likely due to the effective blockage of the HER sites on the GDE by the deposited NCs (for the surface area calculations, refer to Note S1, Supporting Information).

It is worth noting that the ClAg_14_ NC loading of 106 nmol cm^−^^2^ (equivalent to 0.28 mg cm^−2^) that exhibited maximum activity was still significantly lower than the typical loading of commercial AgNPs (4.8 mg cm^−2^).^[^ ^8^ ^]^ To gain perspective, the mass of ClAg_14_ NCs produced through the large‐scale synthesis (12.9 g) can catalyze the conversion of ≈180 kg of CO_2_ into CO per day, utilizing an NC loading of 106 nmol cm^−2^ ClAg_14_/GDE at 200 mA cm^−2^.

### 3.4 DFT Calculations

To gain further insight into the exceptional CO_2_RR activity and selectivity exhibited by the ClAg_14_ NCs, we conducted computational calculations to determine the free energies of the CO_2_RR and HER intermediates. The alkynyl ligands (C≡C^t^Bu) were replaced with C≡CCH_3_ ligands to save computational costs. The DFT calculations were performed on the intact NCs and on the NCs with alkynyl ligand removed, allowing for a comparison. The charge state of the intact ClAg_14_ NCs was found to be +1 at zero applied potential (Figure S17, Supporting Information). We assumed that the ligand‐removed NCs have the same charge state. Details of the DFT calculations can be found in the Experimental Section.

**Figure 4:**
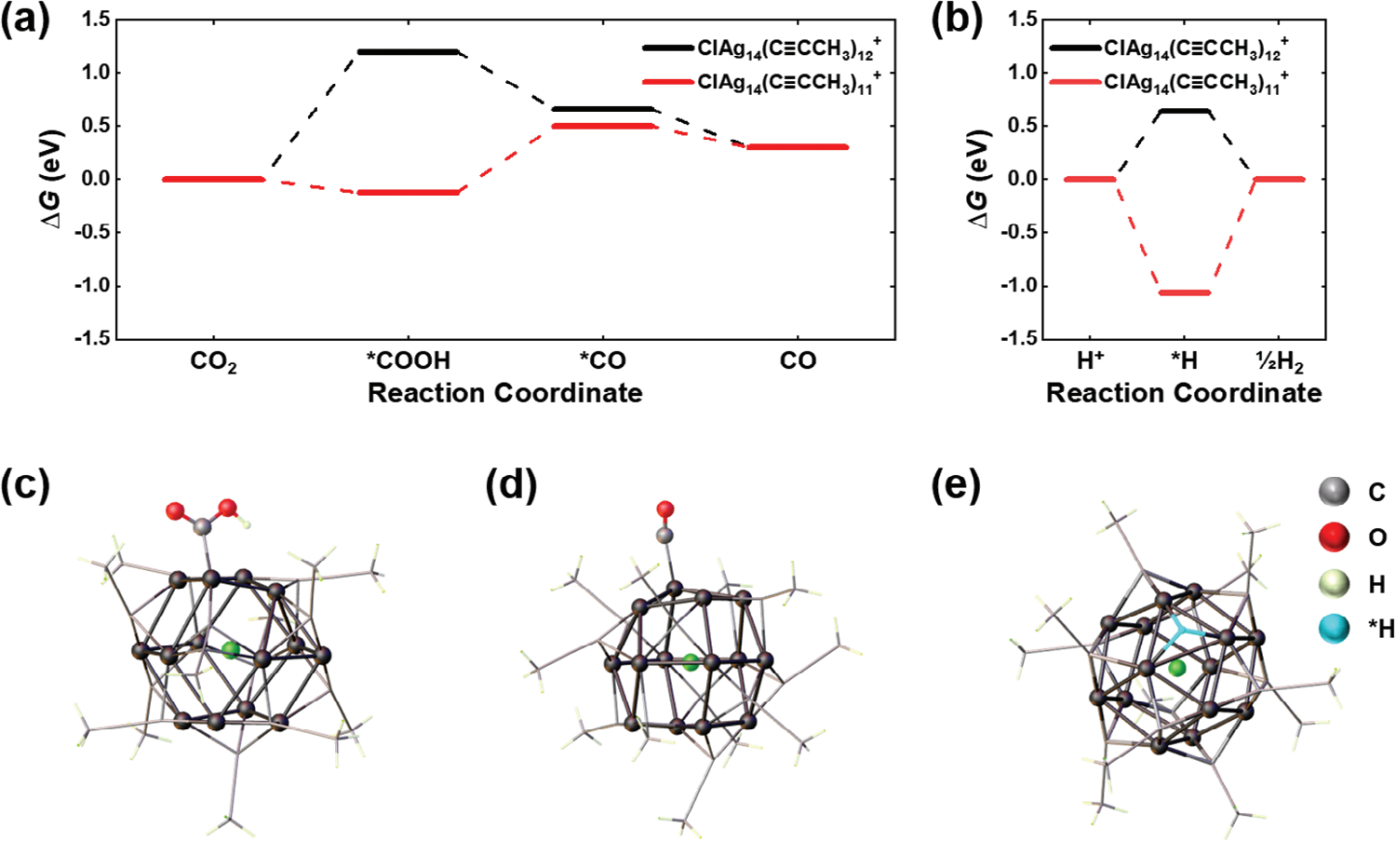
Free energy (Δ*G*) diagrams of the a) CO_2_‐to‐CO and b) H^+^‐to‐½H_2_ conversions on ClAg_14_(C≡CCH_3_)_12_^+^ and ClAg_14_(C≡CCH_3_)_11_^+^ NCs at zero applied potential. Optimized structures of the c) *COOH‐, d) *CO‐, and e) *H‐adsorbed ClAg_14_(C≡CCH_3_)_11_^+^ NCs (color code as in Figure 1; alkyl chains are shown in wireframe form for clarity).

**Figure** **4**a depicts the free energy diagrams for the CO_2_RR intermediates produced on the intact ClAg_14_(C≡CCH_3_)_12_^+^ and ClAg_14_(C≡CCH_3_)_11_^+^ NCs, which serve as models for the de‐ligated NC at zero applied potential. The elemental step that exhibits the maximum change in free energy is defined as the PDS of the reaction. On the intact ClAg_14_(C≡CCH_3_)_12_^+^ NCs, the formation of *COOH intermediate is predicted to have the highest energy barrier, with a reaction energy of 1.20 eV. However, when a single ligand is removed, the thermodynamic energy barrier for the formation of the *COOH intermediate is dramatically reduced to near zero. Consequently, the *CO formation step becomes the PDS with a reaction energy of 0.62 eV, suggesting that the de‐ligation significantly facilitates the conversion of CO_2_ to CO. These computational results were consistent with the experimental results, which indicated that the de‐ligated NC was the catalytically active form (Figure 2) and that the second electron‐transfer step was the PDS of the CO_2_RR (Figure 3b). Furthermore, we performed computations to determine the free energies of the competing HER process and compared them with those of the CO_2_RR. The free energy diagram for the H^+^‐to‐½H_2_ reduction reaction shows that the formation of the *H intermediate is highly exergonic (−1.06 eV) on the de‐ligated ClAg_14_(C≡CCH_3_)_11_^+^ NC, while on the intact ClAg_14_(C≡CCH_3_)_12_^+^ NC, it is endergonic (+0.64 eV) (Figure 4b).

Comparing the CO_2_RR and HER processes, the *COOH formation step is significantly more endergonic compared to the *H formation step on intact cluster, with a difference of 0.56 eV. This suggests that it is more energetically favorable to generate H_2_ rather than CO on the intact ClAg_14_(C≡CCH_3_)_12_^+^ NCs. Conversely, for the de‐ligated ClAg_14_(C≡CCH_3_)_11_^+^ NCs, the PDS of the CO_2_RR is the *CO formation step, while the PDS of the HER process is the H_2_‐releasing step. The reaction energy for the former is lower than the latter by 0.44 eV, which supports the experimental observation of high CO selectivity for the de‐ligated ClAg_14_ NCs (Figure 3a). The optimized adsorption geometries of the reaction intermediates are illustrated in Figure 4c–e and Figure S18 (Supporting Information). Both intact and de‐ligated NCs exhibited the Ag sites within the octahedral Ag_6_ cage as the active sites for CO_2_‐to‐CO conversion (Figures 4c,d; Figure S18a,b, Supporting Information). However, the undercoordinated Ag active site in the de‐ligated ClAg_14_(C≡CCH_3_)_11_^+^ NCs exhibits a stronger binding affinity for the CO_2_RR intermediates, as evident by the significantly decreased Ag─C bond length in adsorbed *COOH and *CO (2.116 and 2.124 Å, respectively, Figure 4c,d) compared to those in the intact ClAg_14_(C≡CCH_3_)_12_^+^ NCs (2.207 and 2.717 Å, respectively, Figure S18a,b, Supporting Information).

The active site in the de‐ligated ClAg_14_(C≡CCH_3_)_11_^+^ NCs effectively lowers the energy barrier for the CO_2_RR intermediates, facilitating the conversion of CO_2_ to CO. Conversely, in the intact ClAg_14_(C≡CCH_3_)_12_^+^ NCs, the *H intermediate is predicted to adsorb at the same active site, competing with the CO_2_RR. (Figure S18c, Supporting Information). However, the *H tends to strongly adsorb on the three‐fold hollow site of the Ag_3_ triangle site exposed by ligand removal on the ClAg_14_(C≡CCH_3_)_11_^+^ NCs (Figure 4e). Consequently, the *H desorption required to form H_2_ becomes the PDS with a high energy barrier. Based on these findings, we can conclude that the presence of different active sites exhibiting different binding properties for the CO_2_RR and HER intermediates is a unique characteristic of the de‐ligated ClAg_14_(C≡CCH_3_)_11_^+^ NCs, which provides a clear explanation for the exceptional CO selectivity observed in the system.

The changes in the adsorption properties observed in the de‐ligated ClAg_14_(C≡CCH_3_)_11_^+^ NCs can be further understood by considering the influence of electronic structures on the adsorption strengths of adsorbates on metal surfaces. It is well‐known that the metal‐adsorbate interactions are strongly dependent on the electronic structures,^[^ ^40^ ^]^ particularly the upshift of *d*‐states, which leads to enhanced binding strength.^[^ ^41^ ^]^ This effect has been observed in various catalytic systems. In the case of the CO_2_RR, it has been established that the metal surfaces with the optimal energy levels of *d*‐states exhibit high catalytic activity. The projected density of states (PDOS) calculations showed that the energy of *d*‐states of the staple Ag site was significantly upshifted from −3.24 eV (relative to the Fermi level) for the intact ClAg_14_(C≡CCH_3_)_12_^+^ NCs to −2.03 eV for the de‐ligated ClAg_14_(C≡CCH_3_)_11_^+^ NCs (Figure S19, Supporting Information). It can be concluded that the upshifted energy of the *d*‐state in the Ag active site provides an appropriate binding strength for the CO_2_ intermediates, leading to exceptional catalytic activity for the CO_2_‐to‐CO conversion.

### 3.5 CO_2_RR Performance in a Zero‐Gap Cell

**Figure 5:**
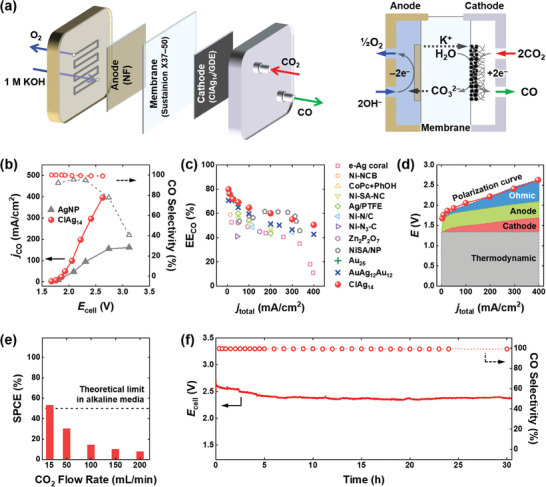
a) Schematic of a zero‐gap CO_2_ electrolyzer configuration (left) and detailed reaction trace scheme (right). b) *j*_CO_ and CO selectivity measured in ClAg_14_/GDE‐ and AgNP/GDE‐equipped zero‐gap CO_2_ electrolyzers as functions of the full‐cell potential (*E*_cell_). The loadings of the ClAg_14_ and Ag NP on GDE were equal to 0.28 and 4.8 mg cm^−2^, respectively. c) Comparison of EE_CO_ measured on the ClAg_14_/GDE in the zero‐gap CO_2_ electrolyzer with those measured on other electrocatalysts. d) Polarization curve of the ClAg_14_/GDE‐equipped zero‐gap cell and the voltage breakdowns. e) SPCE was measured on ClAg_14_/GDE (5 cm^2^) at a current density of 400 mA cm^−2^ with different CO_2_ flow rates. The dashed line indicates the theoretical limit under alkaline media. f) Long‐term CO_2_‐to‐CO electrolysis performed on ClAg_14_/GDE‐equipped zero‐gap cell. The cell performance was evaluated by *E*_cell_ and CO selectivity at a current density of 200 mA cm^−^^2^ with a flowing 0.5 m KOH anolyte solution. In panels (b) and (c), the errors lie within the symbols in the graphs.

To evaluate the practical applicability of the ClAg_14_ NCs, we conducted CO_2_‐to‐CO conversion experiments using a zero‐gap cell (**Figure** **5**a) that is considered a commercially relevant electrolyzer design offering low cell resistance.^[^ ^42^ ^]^ ClAg_14_ NC‐coated GDE with an NC loading of 106 nmol cm^−2^ (0.28 mg cm^−2^) was used for these experiments based on the optimal loading determined in Figure 3d. The setup included an NC‐loaded GDE and an NF as cathode and anode, respectively, and an AEM (Sustainion X37‐50, RT grade, Dioxide Materials) positioned between the electrodes. During the experiments, humidified CO_2_ gas was supplied to the cathode side, while a 1.0 m KOH electrolyte solution was fed to the anode side (details in the Experimental Section).

Figure 5b illustrates the CO_2_RR activities of AgNP/GDE and ClAg_14_/GDE in the zero‐gap cell at various cell potentials (*E*_cell_). The ClAg_14_ NCs consistently exhibited higher CO_2_RR activity compared to the AgNP across all cell potentials. The onset of CO formation was observed at 1.68 V in the ClAg_14_ NC‐equipped zero‐gap cell, and the *j*_CO_ value exhibited an exponential increase with increasing cell potential. At an *E*_cell_ of 2.63 V (without iR compensation), the *j*_CO_ value reached 400 mA cm^−2^, which is considerably low compared to zero‐gap cells employing other efficient catalysts.^[^ ^4^ ^]^ It is worth noting that the *j*_CO_ value of 400 mA cm^−2^ was achieved with a ClAg_14_ loading of 0.28 mg cm^−2^, demonstrating a remarkable catalyst activity of >1400 A g^−1^. Importantly, the CO selectivity remained above 99% across the entire current range for ClAg_14_/GDE, whereas AgNPs experienced a significant decline in CO selectivity when the current density exceeded 100 mA cm^−2^. This decline in CO selectivity was attributed to the enhanced HER occurring on the wetted AgNP/GDE surface at high current densities.^[^ ^43^ ^]^

Cost analysis of the electrochemical CO_2_‐to‐CO conversion process has revealed that the production cost is highly influenced by fluctuations in electricity price cost.^[^ ^44^ ^]^ One key parameter for achieving industrially viable CO_2_RR is energy efficiency for CO production (EE_CO_), which is determined by the FE_CO_ and *E*_cell_ (Equation 8):

(8) $${\mathrm{EE}}_{\mathrm{CO}}=\frac{1.34\;V}{\left|E_{\mathrm{cell}}\right|}\times {\mathrm{FE}}_{\mathrm{CO}}$$

where 1.34 V is a thermodynamic potential gap between CO_2_‐to‐CO conversion and oxygen evolution reaction. Figure 5c and Table S3 (Supporting Information) provide a comparison of EE_CO_ achieved with the ClAg_14_ NCs and other electrocatalysts. As shown in the figure, the EE_CO_ values of the ClAg_14_ NCs were comparable to those of the state‐of‐the‐art electrocatalysts.^[^ ^4^ ^]^ The ClAg_14_/GDE‐equipped zero‐gap cell demonstrated exceptional EE_CO_ of 60% at a commercially relevant current density of 200 mA cm^−2^. Moreover, it exhibited an EE_CO_ value of 51% even at 400 mA cm^−2^, surpassing any reported EE_CO_ values measured at 400 mA cm^−2^ to the best of our knowledge.

To understand the polarization losses that occur during CO_2_‐H_2_O co‐electrolysis in the zero‐gap electrolyzer, we analyzed voltage components. Due to the difficulty of the half‐cell measurement in a zero‐gap cell,^[^ ^45^ ^]^ we separately monitored cathodic and anodic overpotentials, as well as ohmic losses (Figure 3d; Figures S20 and S21 and Note S2, Supporting Information for details). As shown in Figure 5d, the sum of each polarization loss component closely aligned with the experimentally obtained polarization curve in the zero‐gap electrolyzer, suggesting reasonable voltage breakdowns. Notably, the cathodic overpotential contribution from the ClAg_14_ NC/GDE was the smallest among the components, indicating that anode and membrane developments remain future challenges (Figure 5d).

Another crucial system parameter is the SPCE, which represents the ratio of converted CO_2_ to the input CO_2_ in a cell. In alkaline media CO_2_RR, the SPCE is typically limited to 50% due to the neutralization of CO_2_ with OH^−^ ion following the electrochemical CO_2_‐to‐CO conversion (Note S3, Supporting Information for details).^[^ ^46^ ^]^ The highly efficient and selective CO_2_‐to‐CO conversion catalyzed by the ClAg_14_/GDE motivated us to monitor the SPCE at 400 mA cm^−2^ with varying CO_2_ flow rates. As illustrated in Figure 5e, the SPCE exponentially increased as the CO_2_ feeding rate decreased. At a flow rate of 15 mL min^−1^, a maximum SPCE of 53% was achieved, approaching the theoretical limit in alkaline media.

Finally, the long‐term stability of the ClAg_14_/GDE‐equipped zero‐gap CO_2_ electrolyzer was evaluated by monitoring *E*_cell_ and CO selectivity at 200 mA cm^−2^. A dilute 0.5 m KOH anolyte solution was used to prevent the undesired cation crossover through the anion exchange membrane caused by the concentrated KOH solution.^[^ ^43^ ^]^ Figure 5f shows that the full‐cell potential gradually decreased during the initial 4 h due to the activation of ClAg_14_/GDE and NF electrodes but then stabilized at 2.35 V for 30 h. Importantly, the CO selectivity remained consistently above 99%, even though the ClAg_14_/GDE experienced significant wetting during prolonged operation. These results highlight the high activity and intrinsic selectivity of the ClAg_14_ NCs as a CO_2_‐to‐CO catalyst suitable for commercial CO_2_ electrolysis.

## 4 Conclusion

We have successfully demonstrated the large‐scale synthesis of atomically precise ClAg_14_ NCs, achieving a high yield of 90% on a scale of over 10 g. These ClAg_14_ NCs exhibited remarkable CO_2_RR activity and a CO selectivity exceeding 99%. Postmortem analysis revealed that the ClAg_14_(C≡C^t^Bu)_12_ NC underwent a transformation into the catalytically active ClAg_14_(C≡C^t^Bu)_8_ form by losing its alkynyl ligands under reductive potentials. Mechanistic investigations further revealed that the ligand‐removed ClAg_14_(C≡C^t^Bu)_8_ NCs generated de‐ligated Ag sites and Ag_3_ triangle sites as CO_2_RR and HER active sites, respectively. The exceptional CO_2_RR activity and CO selectivity observed in the ClAg_14_ NCs were attributed to these distinct active sites with unique adsorption properties for reaction intermediates, leading to enhanced CO_2_RR and suppressed hydrogen production. Finally, industrially relevant CO_2_‐to‐CO electroreduction was demonstrated using the ClAg_14_ NCs in a zero‐gap CO_2_ electrolyzer, achieving exceptional EE_CO_ value of 51% and catalyst activity of over 1400 A g^−1^ at 400 mA cm^−2^. We envision that the atomically precise metal NCs offering clear structure‐property relationships can provide useful catalyst design principles for various electrocatalytic CO_2_ conversion processes.

## Conflict of Interest

The authors declare no conflict of interest.

## Supporting information

Supporting Information

## Data Availability

The data that support the findings of this study are available from the corresponding author upon reasonable request.
