# Supplementary material for: ClAg14(C≡CtBu)12 Nanoclusters as Efficient and Selective Electrocatalysts Toward Industrially Relevant CO2 Conversion
Source: Adv Sci (Weinh). 2023 Dec 25;11(10):2306089. doi: 10.1002/advs.202306089 (PMC10933691; doi:10.1002/advs.202306089)
Supplement: Supplementary file 1 — Supporting Information [file ADVS-11-2306089-s001.pdf]

ADVANCED SCIENCE
Open Access

Supporting Information

for *Adv. Sci.*, DOI 10.1002/advs.202306089

$ClAg_{14}(C{\equiv}C^tBu)_{12}$ Nanoclusters as Efficient and Selective Electrocatalysts Toward Industrially Relevant CO_2 Conversion

Hoeun Seong, Kiyoung Chang, Fang Sun, Sojung Lee, Sang Myeong Han, Yujin Kim, Chang Hyuck Choi, Qing Tang* and Dongil Lee**

Supporting Information

$ClAg_{14}(C{\equiv}C^tBu)_{12}$ Nanoclusters as Efficient and Selective Electrocatalysts Toward Industrially Relevant CO_2 Conversion

Hoeun Seong,[†] *Kiyoung Chang,*[†] *Fang Sun,*[†] *Sojung Lee, Sang Myeong Han, Yujin Kim, Chang Hyuck Choi,* Qing Tang,* and Dongil Lee**

[†]These authors contributed equally to this work.
*Corresponding Author's e-mail: chchoi@postech.ac.kr (C.H.C.); qingtang@cqu.edu.cn (Q.T.); dongil@yonsei.ac.kr (D.L.)

Supplementary Note 1: Calculation of the Number of NC layers

We calculated the number of NC layers immobilized in the MPL by comparing the surface area of MPL and NCs. We assumed that the NCs are packed in simple cubic structure, and their ligands are not overlapped.

Brunauer−Emmett−Teller (BET) surface area of the MPL is 30.5 m^2/g, and the weight of MPL is 5.5 mg/cm^2. Thus, the surface area of the MPL per unit geometric area is

$$(30.5\ m^2/g) \times (5.5\ mg/cm^2) = 0.168\ m^2/cm^2.$$

Next, we calculated the cross-section area of one $ClAg_{14}$ NC. The NC diameter is 1.45 nm, including the ligand chains, and therefore the cross-section area of one $ClAg_{14}$ NC is 1.65 x 10^{-18} m^2. Therefore, the mole of NCs required to cover the MPL is

$$(0.168\ m^2/cm^2) / (1.65 \times 10^{-18}\ m^2) / (6.02 \times 10^{23}\ mol^{-1}) = 131\ nmol/cm^2 \text{ for monolayer.}$$

Therefore, 10.6, 53, 106, and 212 $nmol/cm^2$ of $ClAg_{14}$ NC correspond to 0.1, 0.4, 0.8, and 1.6 layers, respectively.

Supplementary Note 2: Voltage Breakdown Analysis and Energy Efficiency

Due to the difficulty in the half-cell measurements of the MEA-based zero-gap electrolyzer, we separately measured the cathodic (η_{cat}) and anodic (η_{an}) overpotentials in a flow electrolyzer and one-component electrochemical cell, respectively.

The η_{cat} measurement was conducted following the electrolyzer setup and method aforementioned. The η_{an} measurement was conducted using a three-electrode system in a one-compartment cell containing 1.0 M KOH electrolyte solution (total volume = 20 ml) with stirring. The NF was used as a working electrode. A Pt foil cathode (1 cm^2) and Ag/AgCl (1.0 M KCl) electrode were used as the counter and reference electrodes, respectively.

Thermodynamic cell potential (ΔE^0) was determined as $|\ E(CO_2/CO) - E(O_2/H_2O)\ | = 1.34$ V.

The applied potentials were measured against a reference electrode, Ag/AgCl (1.0 M KCl). Applied potentials measured against the Ag/AgCl electrode ($E_{Ag/AgCl}$) were converted to the RHE scale (E_{RHE}), and η_{cat} and η_{an} using the following equations (Equation S1–S3):

$$E_{RHE} = E_{Ag/AgCl} + 0.21 + 0.059\ pH, \quad (S1)$$
$$\eta_{cat} = |\ E_{RHE} - E(CO_2/CO)\ |, \quad (S2)$$
$$\eta_{an} = |\ E_{RHE} - E(O_2/H_2O)\ |. \quad (S3)$$

The polarization loss from ohmic drop was calculated by multiplying total current and cell resistance, which was measured using EIS experiments in a zero-gap electrolyzer.

Based on the full-cell potential, The energy efficiency for CO production (EE_{CO}) was calculated using the following equation:

$$EE_{CO}\ (\%) = \Delta E^0 / E_{cell} \text{ x } FE_{CO}, \quad (S4)$$

where E_{cell} is the operating potential obtained in the zero-gap electrolyzer, and FE_{CO} is Faradaic efficiency for CO production.

Supplementary Note 3: Single-Pass Conversion Efficiency (SPCE)

The SPCE of CO_2 was obtained from the equation below:

$$SPCE\ (\%) = \frac{i_{CO}/nF}{v/60RT}, \quad (S5)$$

where i_{CO} is CO partial current (mA), n is the number of electrons required for reaction (2 for CO production), v is the CO_2 flow rate (ml/min), and other symbols are as commonly known.

The neutralization of CO_2 with OH^- ion, a non-electrochemical reaction, follows the electrochemical CO_2-to-CO conversion in neutral-alkaline media as shown below:

$$CO_2 + 2H_2O + 2e^- \rightarrow CO + 2OH^-, \quad (S6)$$
$$CO_2 + 2OH^- \rightarrow CO_3^{2-} + H_2O. \quad (S7)$$

In other words, when one CO_2 is electrochemically converted to CO, another CO_2 is consumed in a non-electrochemical pathway producing carbonate ions, rendering the maximum SPCE of 50%. Following Equation S5, the maximum i_{CO} under 30 ml/min of CO_2 flow rate is calculated to be 1.97 A for a 5 cm^2 electrode at 400 mA/cm^2.

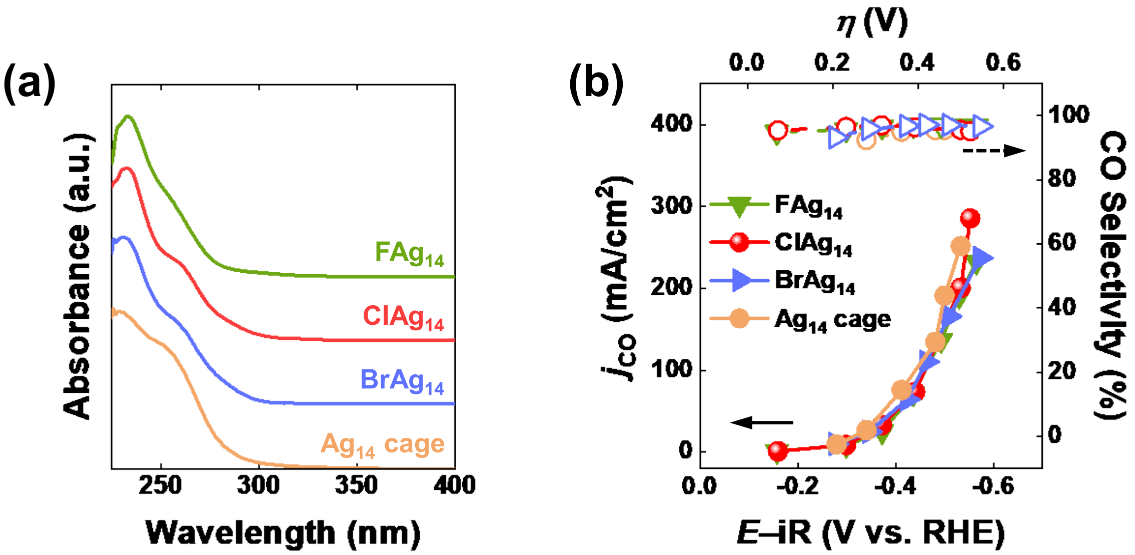

Figure S1. (a) UV-vis absorption spectra and (b) j_{CO} obtained on X@Ag_{14} NCs (X = F, Cl, Br) and hollow Ag_{14} cage.

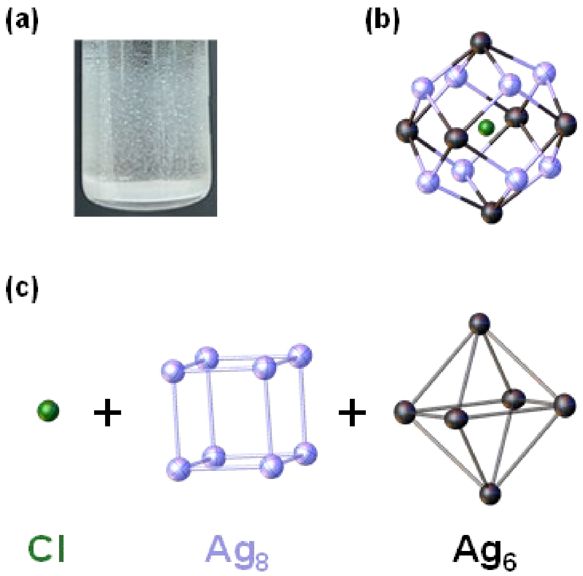

Figure S2. (a) Digital photograph showing single crystals of $[ClAg_{14}(C{\equiv}C^tBu)_{12}]^+[BF_4]^-$ NCs grown at 25 °C by layering diethyl ether over a CH_2Cl_2 NC solution. (b) Core structure of the $ClAg_{14}$ NCs obtained using SC-XRD analysis. (c) Dissecting representations of the core framework.

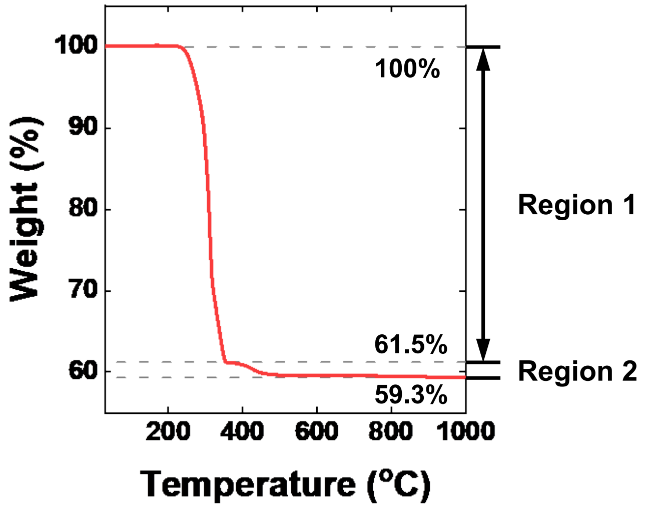

Figure S3. TGA profile of $[ClAg_{14}(C\equiv C^tBu)_{12}]^+[BF_4]^-$ NCs (40 mg). The weight loss of ~41% at 600 °C corresponds to the loss of twelve $C\equiv C^tBu$ alkynyl ligands (Region 1) and the BF_4^- counterion (Region 2).

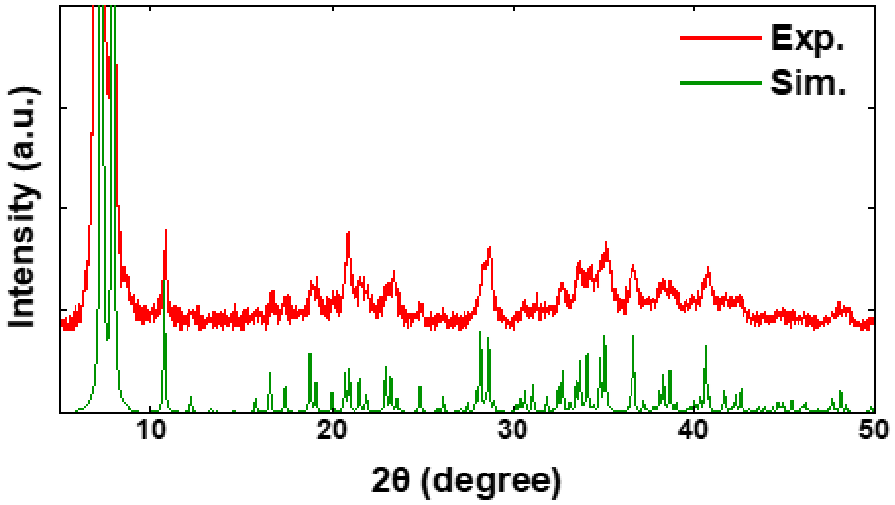

Figure S4. Comparison of the powder XRD pattern of the cluster product synthesized over a 10-gram scale (red line) with the simulated diffractogram based on the crystal structure of $ClAg_{14}$ NCs (green line).

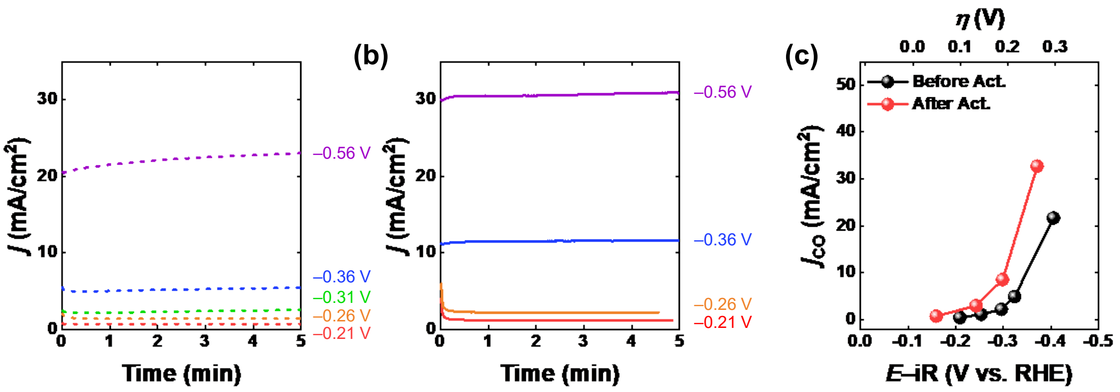

Figure S5. j-time profiles monitored during CPE experiments on (a) pristine and (b) activated $ClAg_{14}$ NC/GDEs in a CO_2-fed flow electrolyzer with a flowing 1.0 M KOH solution. The current monitored at –0.56 V on the pristine $ClAg_{14}$/GDE gradually increased due to the electrochemical activation of the NCs. The current responses monitored on the activated NC/GDE rapidly stabilized within 5 min. (c) Comparison of j_{CO} measured on $ClAg_{14}$/GDEs at various cathodic potentials before and after electrochemical activation. The potentials in panel (c) were iR-corrected.

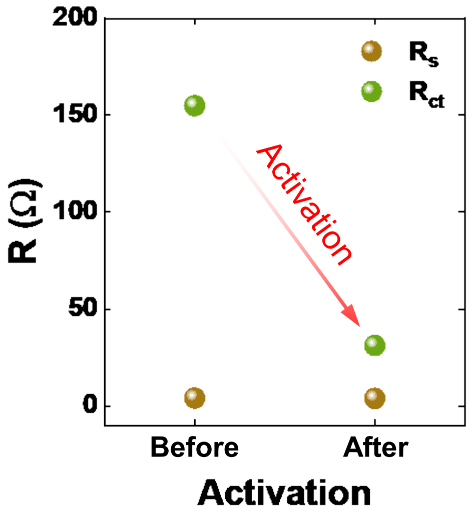

Figure S6. Resistance (R) components obtained from fitting of the Nyquist plots in Figure 2b. R_s, solution resistance; R_{ct}, charge-transfer resistance. R_s value remains unchanged, but R_{ct} value largely decreased with activation.

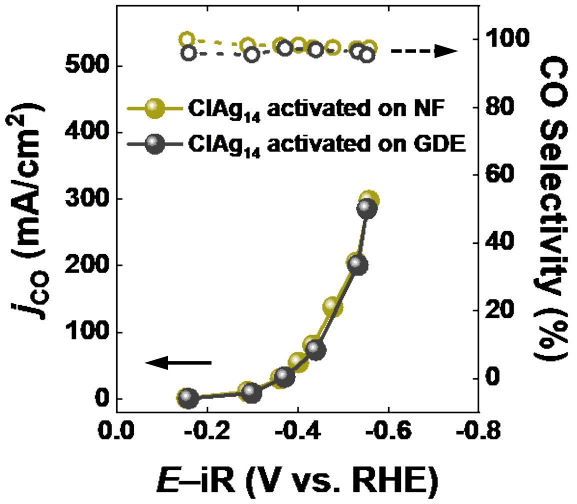

Figure S7. Comparison of j_{CO} and CO selectivity measured on $ClAg_{14}$ NCs activated on a NF (at –0.96 V vs. RHE for 1 h) with those of the $ClAg_{14}$/GDE activated at –0.96 V vs. RHE for 1 h. $ClAg_{14}$ NCs activated on the NF was retrieved from the NF and dropcast on a GDE to measure their CO_2RR activity without further activation process. The potentials were iR-corrected.

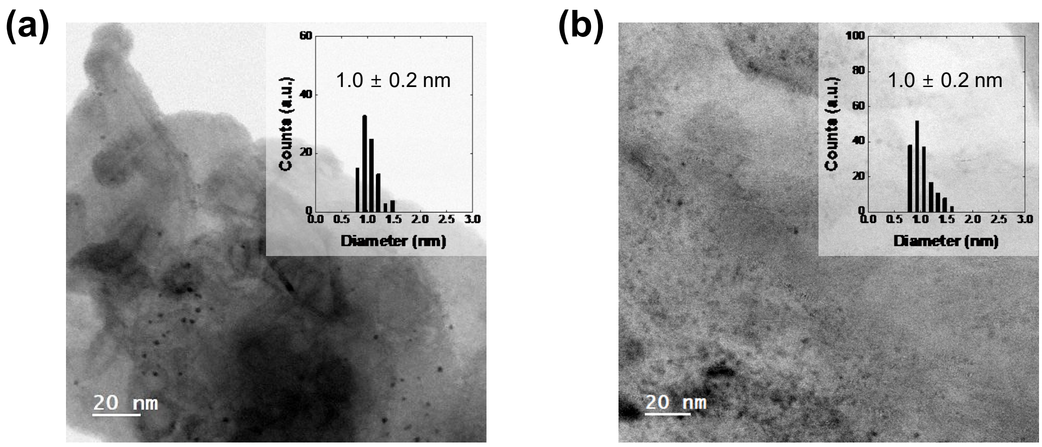

Figure S8. Representative TEM images of $ClAg_{14}$/GDE before and after electrochemical activation at –0.96 V for 1 h. The insets show histograms of core diameters measured in several TEM images. The average diameters were determined to be 1.0 ± 0.2 nm for both $ClAg_{14}$ NCs before and after activation.

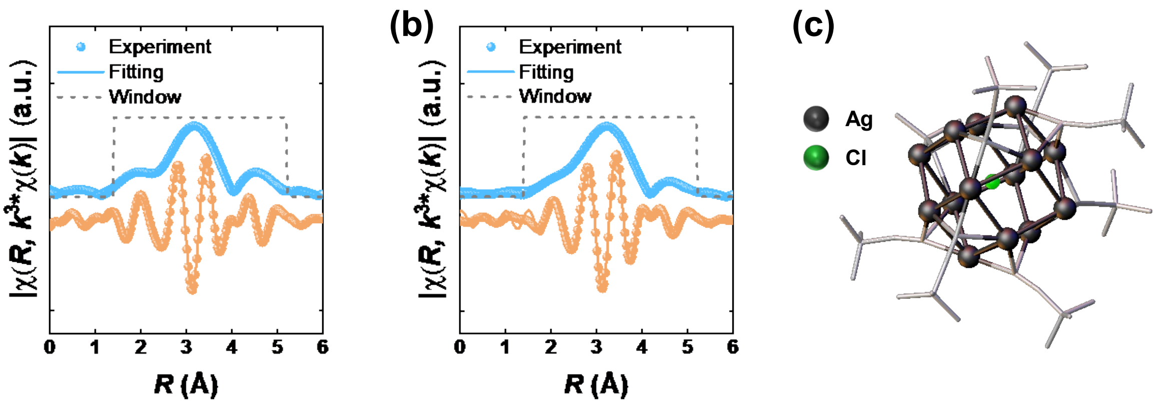

Figure S9. Representative fitted *R* space (azure) and *k* space (orange) Ag K-edge EXAFS spectra of the (a) pristine and (b) activated $ClAg_{14}$ NCs. (c) The optimized structure of the $ClAg_{14}$ NCs after losing four ligands. Alkyl chains are shown in wireframe.

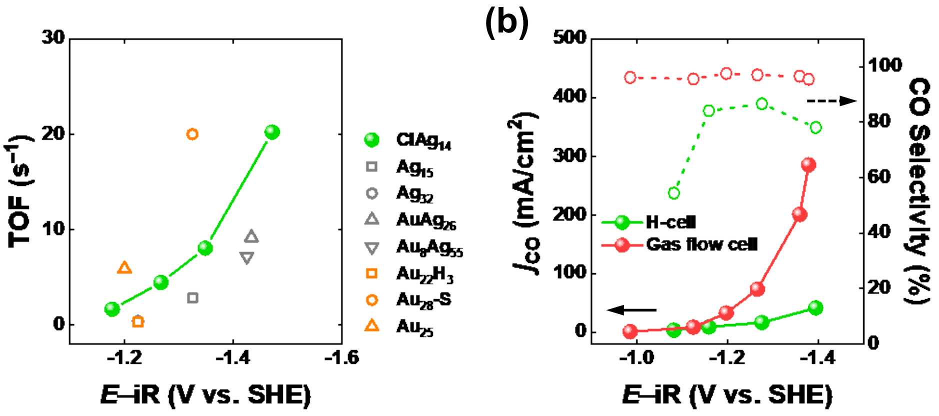

Figure S10. (a) Comparison of turnover frequency (TOF) values of various Ag- and Au-based NCs measured in H-cell electrolyzers. The NC loadings were in the range of 0.03-0.5 mg/cm^2 [1] and the $ClAg_{14}$ loading was 0.28 mg/cm^2 (10.6 nmol/cm^2). (b) Comparison of j_{CO} and CO selectivity values of $ClAg_{14}$/GDE measured in H-cell and gas flow cell. pH-independent standard hydrogen electrode (SHE) scale was used to compare data measured in various pH conditions. The potentials were iR-corrected.

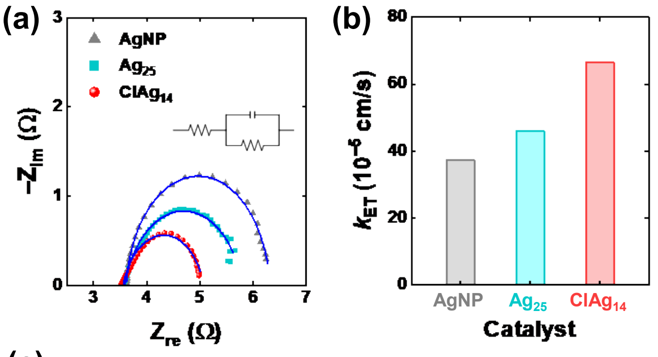

(c)

Catalyst	R_s (Ω)	R_{ct} (Ω)	k_{ET} (10^{-5} cm/s)
AgNP	3.6	2.7	37
Ag_{25}	3.6	2.2	46
$ClAg_{14}$	3.6	1.5	67

Figure S11. (a) Nyquist plots and (b) electron-transfer rate constant (k_{ET}) measured at –0.56 V vs. RHE on the AgNP/GDE, Ag_{25}/GDE, and $ClAg_{14}$/GDE in a CO_2-fed flow electrolyzer with flowing 1.0 M KOH electrolyte solution. (c) Resistance parameters and k_{ET} values obtained from fitting. The equivalent electric circuit[2] used to fit the electrochemical impedance spectra is shown in the inset of (a). R_s, solution resistance; R_{ct}, charge-transfer resistance; Q_{dl}, constant phase element for double layer.

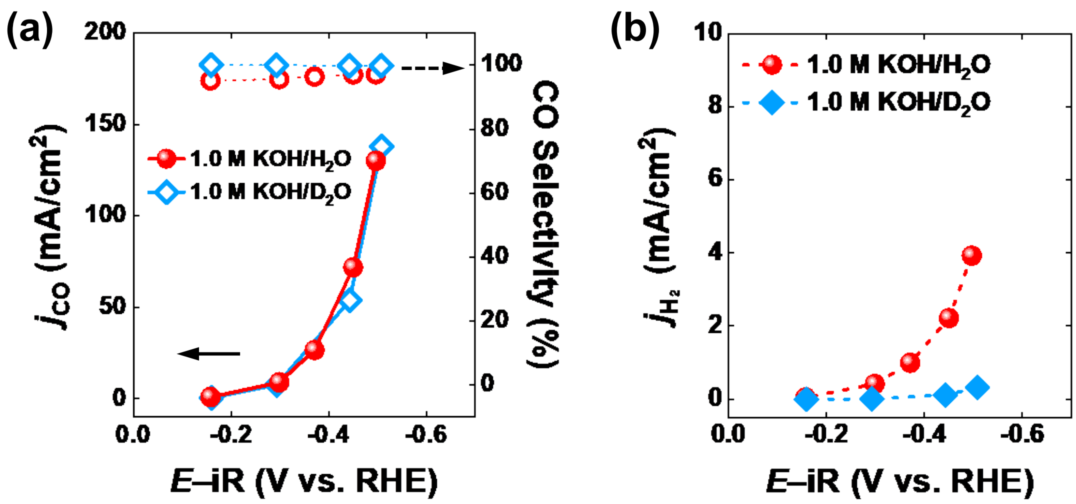

Figure S12. (a) j_{CO}, CO selectivity, and (b) j_{H_2} values obtained in H_2O- and D_2O-based 1.0 M KOH electrolyte solutions on $ClAg_{14}$ NCs during CO_2RR. The potentials were iR-corrected.

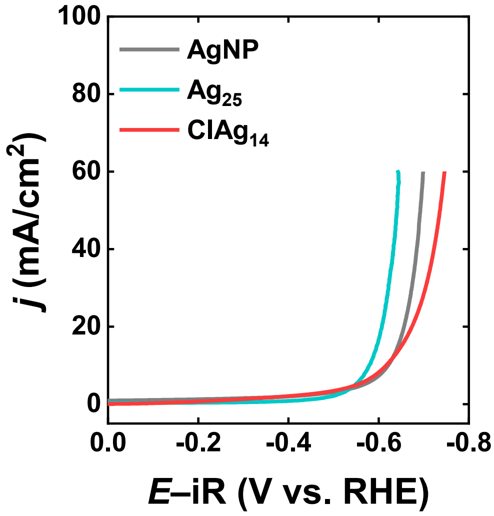

Figure S13. LSV traces measured on various Ag catalysts in the Ar-fed flow electrolyzer with a flowing 1.0 M KOH electrolyte solution. The potentials were iR-corrected.

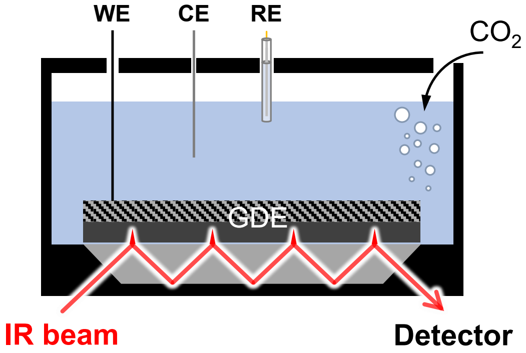

Figure S14. Schematic of the operando ATR-FTIR experimental setup. The electrochemical cell was filled with 1.0 M $NaClO_4$ electrolyte solution and the solution was purged with CO_2 gas for 30 min before conducting operando experiments. The CO_2 gas was kept blowing to the headspace of the cell during the experiments. The activated $ClAg_{14}$/GDE was tightly mounted on the ATR crystal with the MPL side facing down the crystal, allowing a very thin layer of electrolyte solution between them.

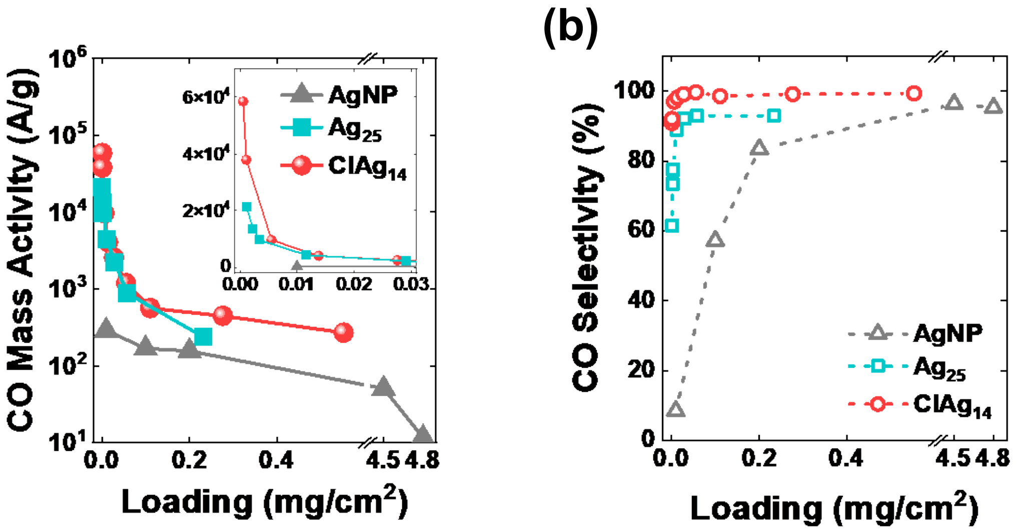

Figure S15. (a) CO mass activity and (b) CO selectivity measured at –0.96 V vs. RHE for Ag-based catalysts as functions of catalyst loading. The j_{CO} of $ClAg_{14}$/GDE linearly increased with increasing the NC loading and levelled off at the loading of over 106 nmol/cm^2 (0.28 mg/cm^2). Since the j_{CO} value levelled off at higher loading, the CO_2RR activities were evaluated up to their levelling points; 0.56 mg/cm^2 and 0.23 mg/cm^2 for the $ClAg_{14}$/GDE and Ag_{25}/GDE, respectively.

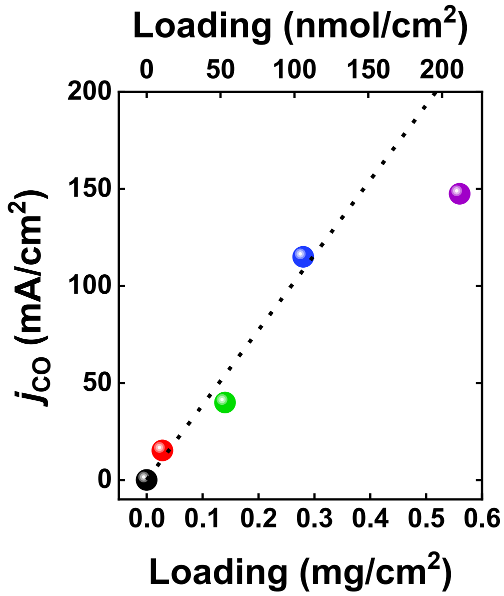

Figure S16. j_{CO} values of $ClAg_{14}$/GDE at –0.3 V vs. RHE (iR-corrected) at different NC loadings.

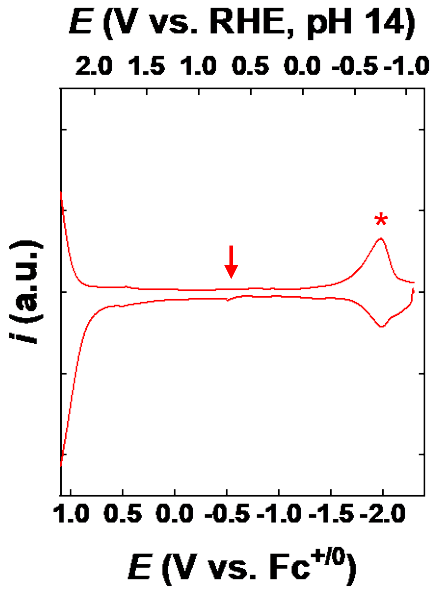

Figure S17. Square-wave voltammogram of $ClAg_{14}$ NCs in CH_2Cl_2 solution containing 0.1 M Bu_4NPF_6. The arrow denotes the open circuit potential (OCP) of the NC solution. The asterisk denotes the first reduction peak of $ClAg_{14}$ NC. Comparison of the formal potentials of $ClAg_{14}$ NCs with the CO_2-to-CO reduction potential in RHE scale shows that $ClAg_{14}$ NCs maintain their initial charge state of +1 until their first reduction potential of –0.7 V vs. RHE.

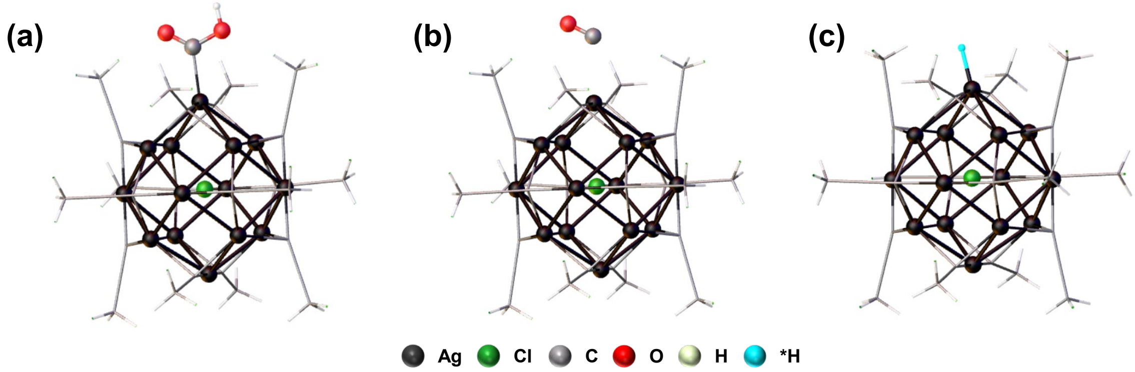

Figure S18. Optimized structures of (a) *COOH-, (b)*CO-, and (c) *H-adsorbed $ClAg_{14}(C{\equiv}CCH_3)_{12}^+$ NCs.

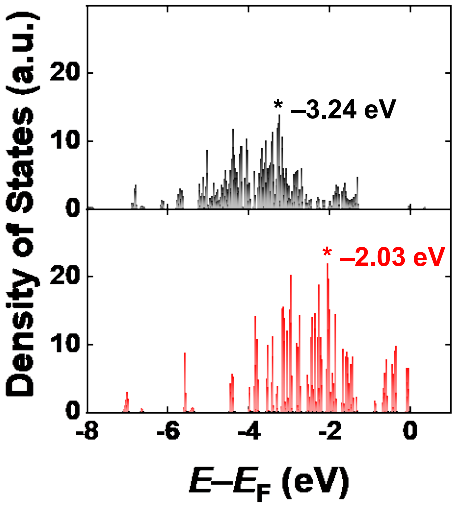

Figure S19. Projected density of states of the *d*-states of the Ag active sites in the $ClAg_{14}(C{\equiv}CCH_3)_{12}^+$ (black) and $ClAg_{14}(C{\equiv}CCH_3)_{11}^+$ (red) NCs.

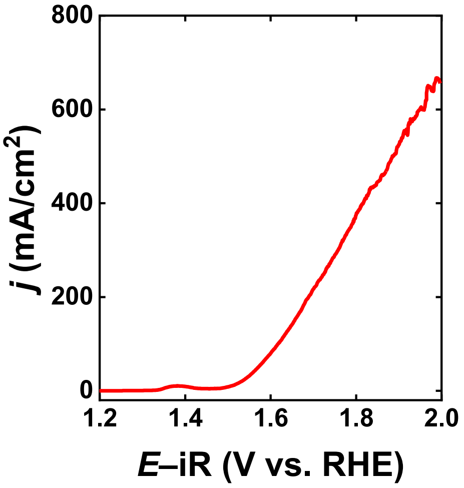

Figure S20. LSV traces recorded at 20 mV/s on the NF electrode in a one-compartment three-electrode cell containing 1.0 M KOH electrolyte solution. The potentials were iR-corrected.

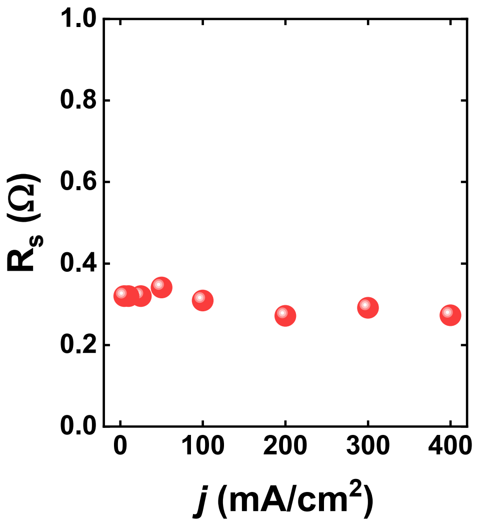

Figure S21. R_s values measured in a zero-gap CO_2 electrolyzer with a flowing 1.0 M KOH anolyte solution. Of note, the R_s values are very low compared to those measured in the gas flow cell (~3.6 Ω, Figure S10).

Table S1. Summary of the Ag K-edge fitting results of EXAFS for pristine and activated $ClAg_{14}$ NCs.

Sample	Scattering path[a]	CN[b]	R (Å)[c]	σ^2 (Å)[c]	R-factor
**Pristine $ClAg_{14}$**	Ag_{octa}–C	0.86 [12/14]	2.11 (0)	0.002 (1)	0.005
	Ag_{cubic}–C	1.71 [24/14]	2.40 (0)	0.003 (0)	
	Ag_{cubic}–Ag_{octa}	3.43 [48/14]	2.80 (0)	0.020 (1)	
	Ag_{cubic}–Ag_{cubic}	1.71 [24/14]	3.67 (1)	0.007 (1)	
	Ag–Cl	1.00	3.39 (0)	0.002 (1)	
**Activated $ClAg_{14}$**	Ag_{octa}–C	0.57 [8/14]	1.83 (1)	0.007 (2)	0.009
	Ag_{cubic}–C	1.14 [16/14]	2.76 (1)	0.003 (1)	
	Ag_{cubic}–Ag_{octa}	3.43 [48/14]	2.69 (0)	0.010 (0)	
	Ag_{cubic}–Ag_{cubic}	1.71 [24/14]	3.54 (2)	0.037 (3)	
	Ag–Cl	1.00	3.38 (0)	0.002 (0)	

[a] Ag_{octa} and Ag_{cubic} denote octahedral Ag_6 and cubic Ag_8, respectively.
[b] The values in square brackets are fractional expressions of each value.
[c] The values in parenthesis are standard deviations.

Table S2. Benchmarks of CO_2RR performance of metal NCs in H-cells

Cathode Catalyst	Synthetic Yield (%)	Electrolyte	Potential (V vs. SHE)	FE_{CO} (%)	j_{CO} (mA/cm^2)	TOF (s^{-1})	Refs.
**$ClAg_{14}$**	90	0.05 M $KHCO_3$ + 1.0 M KCl	-1.35	87	16	8	This Work
**Ag_{15}**	9	0.5 M $KHCO_3$	–1.32	90	13	2.8	[3]
**Ag_{32}**	14	0.5 M $KHCO_3$	–1.22	96	6	0.4	[4]
**$AuAg_{26}$**	not available	EMIM-BF_4/H_2O (V/V=7/1)	–1.43	94	12	9.2	[5]
**Au_8Ag_{55}**	41	0.5 M $KHCO_3$	–1.43	65	21	7.2	[6]
**$Au_{22}H_3$**	35	0.5 M $KHCO_3$	–1.22	72	11	0.3	[7]
**Au_{28}–S**	very low	0.5 M $KHCO_3$	–1.32	98	7	20	[8]
**Au_{25}**	50[9]	0.1 M $KHCO_3$ + 0.4 M KCl	–1.2	78	12	5.9	[10]

Table S3. Benchmarks of energy efficiency for CO production (EE_{CO})

Cathode Catalyst	Electrolyte	*j* (mA/cm^2)	EE_{CO} (%)	Refs.
**$ClAg_{14}$**	1 M KOH	200	60	This Work
		400	51	
**e-Ag coral**	1 M KOH	260	41	[11]
**Ni-NCB**	0.5 M $KHCO_3$	112	50	[12]
**CoPc + PhOH**	1 M KOH	200	47	[13]
**Ni-SA-NC**	0.5 M $KHCO_3$	200	48	[14]
**Ag/PTFE**	1 M KOH	200	44	[15]
**Ni-N/C**	0.1 M $KHCO_3$	300	45	[16]
**$Ni-N_3-C$**	1 M KOH	45	41	[17]
**$Zn_2P_2O_7$**	1 M KOH	150	45	[18]
**NiSA/NP**	1 M KOH	234	61	[19]
**Au_{25}**	1 M KOH	100	64	[20]
**$AuAg_{12}Au_{12}$**	1 M KOH	240	50	[21]

References

[1] O. M. Abu-Salah, M. H. Ja'far, A. R. Al-Ohaly, K. A. Al-Farhan, H. S. Al-Enzi, O. V. Dolomanov, J. A. K. Howard, *Eur. J. Inorg. Chem.* **2006**, *2006*, 2353-2356.
[2] M. Asadi, K. Kim, C. Liu, A. V. Addepalli, P. Abbasi, P. Yasaei, P. Phillips, A. Behranginia, J. M. Cerrato, R. Haasch, P. Zapol, B. Kumar, R. F. Klie, J. Abiade, L. A. Curtiss, A. Salehi-Khojin, *Science* **2016**, *353*, 467-470.
[3] L. Qin, F. Sun, X. Ma, G. Ma, Y. Tang, L. Wang, Q. Tang, R. Jin, Z. Tang, *Angew. Chem. Int. Ed.* **2021**, *60*, 26136-26141.
[4] L. Chen, F. Sun, Q. Shen, L. Qin, Y. Liu, L. Qiao, Q. Tang, L. Wang, Z. Tang, *Nano Res.* **2022**, *15*, 8908-8913.
[5] X. Lin, W. Ma, K. Sun, B. Sun, X. Fu, X. Ren, C. Liu, J. Huang, *J. Phys. Chem. Lett.* **2021**, *12*, 552-557.
[6] J. Hu, M. Zhou, K. Li, A. Yao, Y. Wang, Q. Zhu, Y. Zhou, L. Huang, Y. Pei, Y. Du, S. Jin, M. Zhu, *Small* **2023**, 2301357.
[7] Z.-H. Gao, K. Wei, T. Wu, J. Dong, D.-e. Jiang, S. Sun, L.-S. Wang, *J. Am. Chem. Soc.* **2022**, *144*, 5258-5262.
[8] J. Wang, F. Xu, Z.-Y. Wang, S.-Q. Zang, T. C. W. Mak, *Angew. Chem. Int. Ed.* **2022**, *61*, e202207492.
[9] M. Zhu, E. Lanni, N. Garg, M. E. Bier, R. Jin, *J. Am. Chem. Soc.* **2008**, *130*, 1138-1139.
[10] W. Choi, H. Seong, V. Efremov, Y. Lee, S. Im, D.-H. Lim, J. S. Yoo, D. Lee, *J. Chem. Phys.* **2021**, *155*, 014305.
[11] W. H. Lee, Y.-J. Ko, Y. Choi, S. Y. Lee, C. H. Choi, Y. J. Hwang, B. K. Min, P. Strasser, H.-S. Oh, *Nano Energy* **2020**, *76*, 105030.
[12] T. Zheng, K. Jiang, N. Ta, Y. Hu, J. Zeng, J. Liu, H. Wang, *Joule* **2019**, *3*, 265-278.
[13] S. Ren, D. Joulié, D. Salvatore, K. Torbensen, M. Wang, M. Robert, C. P. Berlinguette, *Science* **2019**, *365*, 367-369.
[14] H.-Y. Jeong, M. Balamurugan, V. S. K. Choutipalli, E.-s. Jeong, V. Subramanian, U. Sim, K. T. Nam, *J. Mater. Chem. A* **2019**, *7*, 10651-10661.
[15] A. Reyes, R. P. Jansonius, B. A. W. Mowbray, Y. Cao, D. G. Wheeler, J. Chau, D. J. Dvorak, C. P. Berlinguette, *ACS Energy Lett.* **2020**, *5*, 1612-1618.
[16] D. Kim, W. Choi, H. W. Lee, S. Y. Lee, Y. Choi, D. K. Lee, W. Kim, J. Na, U. Lee, Y. J. Hwang, D. H. Won, *ACS Energy Lett.* **2021**, *6*, 3488-3495.

[17] W. Hua, H. Sun, L. Lin, Q. Mu, B. Yang, Y. Su, H. Wu, F. Lyu, J. Zhong, Z. Deng, Y. Peng, *Chem. Eng. J.* **2022**, *446*, 137296.

[18] X. Y. Zhang, W. J. Li, J. Chen, X. F. Wu, Y. W. Liu, F. Mao, H. Y. Yuan, M. Zhu, S. Dai, H. F. Wang, P. Hu, C. Sun, P. F. Liu, H. G. Yang, *Angew. Chem. Int. Ed.* **2022**, *61*, e202202298.

[19] W. Ren, X. Tan, C. Jia, A. Krammer, Q. Sun, J. Qu, S. C. Smith, A. Schueler, X. Hu, C. Zhao, *Angew. Chem. Int. Ed.* **2022**, *61*, e202203335.

[20] H. Seong, J. Kim, K. Chang, H.-w. Kim, W. Choi, D. Lee, *J. Electrochem. Sci. Technol.* **2023**, *14*, 243-251.

[21] H. Seong, M. Choi, S. Park, H.-w. Kim, J. Kim, W. Kim, J. S. Yoo, D. Lee, *ACS Energy Lett.* **2022**, *7*, 4177-4184.
